# Polyphenol intake and depressive symptoms in young adults: evidence from a population-based longitudinal study

**DOI:** 10.1017/S0007114525105886

**Published:** 2026-04-14

**Authors:** Elizabeth Gamage, Melissa M. Lane, Mohammadreza Mohebbi, Mojtaba Lotfaliany, Deborah N. Ashtree, Felice N. Jacka, Rebecca Orr, Adrienne O’Neil, Samantha L. Dawson, Wolfgang Marx

**Affiliations:** 1 Deakin University, IMPACT – the Institute for Mental and Physical Health and Clinical Translation, Food & Mood Centre, School of Medicine, Barwon Health, Geelong, Australia; 2 Biostatistics Unit, Faculty of Health, Deakin University, Geelong, Australia; 3 The Institute for Mental and Physical Health and Clinical Translation, School of Medicine, Deakin University, Geelong, Australia; 4 Centre for Adolescent Health, Murdoch Children’s Research Institute, Melbourne, VIC, Australia; 5 College of Public Health, Medical & Veterinary Sciences, James Cook University, Townsville, QLD, Australia

**Keywords:** Nutritional psychiatry, Polyphenols, Depression, Young adults

## Abstract

Due to the high prevalence of depression among young adults, identifying prevention strategies during young adulthood is crucial. Dietary polyphenols have been associated with depression in older cohorts; however, the association remains unclear, particularly in young adults. This study aimed to assess the prospective association between the intake of total polyphenols, polyphenol classes and polyphenol subclasses with depressive symptoms in young adults. Data from 1484 Raine Study Generation 2 participants (52·7 % female; baseline mean age (sd): 20 (0·5)) at the −20, −22, and −27 year follow-ups (*n* 964, 979 and 1094, respectively), with overlap across follow-ups, were used. Energy-adjusted polyphenol intake was estimated from FFQ data using our expansion of the AUSNUT 2011–13 and Phenol-Explorer to include polyphenol content data and categorised into quartiles. The primary outcome was self-reported depressive symptoms assessed via the twenty-one-item Depression, Anxiety and Stress Scale averaged across the three time points. Linear mixed-effects models were used to assess the association between the polyphenol intake exposures and depressive symptoms. Sociodemographic characteristics and lifestyle- and health-related behaviours were adjusted for. Participants in the highest quartiles for flavonol and hydroxybenzoic acid intake had lower depressive symptoms across time than participants in the lowest quartiles (flavonols (Q4 *v.* Q1 mean difference: −1.38; 95% CI −2.48, −0.28); hydroxybenzoic acids (Q4 *v.* Q1: −1.40; 95% CI −2.53, −0.27). We found no evidence of a highest *v.* lowest association for all other polyphenol categories. Future studies are required to investigate whether increasing polyphenol intake could protect against depression in young adults.

Young adulthood is a time of increasing independence and major life transitions. Many young adults will, for the first time, live independently, navigate the workforce, undertake tertiary education^(1)^ and, for some, experience a depressive episode^(2)^. Further, the Institute for Health Metrics and Evaluation reported that, in 2021, depressive disorders were the second highest contributor to disability adjusted life years in young adults aged 20–24, globally^(3)^. As such, understanding novel strategies for the prevention of depression in young adults is of great public health importance.

Nutritional Psychiatry is an emerging field that explores the role of specific nutrients^(4)^, foods^(5)^ and dietary patterns^(6)^ on mental health. Evidence from clinical trials suggests a causal relationship between diet and depression^(6–9)^. Indeed, interventions focused on improving overall diet quality can reduce depressive symptoms^(7,8)^, particularly in individuals with moderate to severe clinical depression^(6)^ and in young adults with elevated levels of depressive symptoms^(9)^. Additionally, observational studies have demonstrated that healthy dietary patterns are inversely associated with depressive outcomes^(10)^, including in young adults^(11)^.

Polyphenols are natural bioactive compounds found in high concentrations in healthy dietary patterns^(12)^, more specifically in plant and plant-derived foods including fruits, vegetables, legumes, nuts, seeds, herbs and spices, as well as their derivatives such as olive oil, tea, coffee and wine^(13)^. Further, several large epidemiological studies have shown that individuals who consume higher intakes of polyphenols have a reduced risk of several other chronic illnesses associated with inflammation, including CVD, type 2 diabetes, some forms of cancer and neurodegenerative diseases^(14–18)^. This is likely relevant to common mental disorders like depression, given the shared risk pathways, risk factors and aetiology^(19)^. As such, it has been proposed that higher intakes of polyphenols may be protective against depression^(20)^. Indeed, a recent Mendelian randomisation study used circulating hippurate, a biomarker of polyphenol intake, and found lower circulating levels in individuals with depression^(21)^. In addition, several cross-sectional^(22,23)^ and prospective studies^(24,25)^ have demonstrated that higher intakes of some polyphenol classes and subclasses are associated with either reduced depressive symptoms or reduced risk of incident depression. For example, cross-sectional inverse associations have been reported for intakes of anthocyanins, flavanones, flavones and isoflavones and depressive symptoms^(22,23)^ and prospective inverse associations for intakes of flavanones, flavones and flavonols and risk of incident depression^(24)^.

Despite the evidence linking polyphenol intake with depression, all previous observational studies were conducted in cohorts not specific to young adults. Given that (a) the brain continues to mature during young adulthood^(26)^, (b) diet quality is generally decreased in young adulthood^(27,28)^ and (c) dietary improvement reduces symptoms of depression^(29)^, implementing dietary strategies (e.g. increasing polyphenol intake) during this life stage offers a window of opportunity to reduce the prevalence of depression later in life. As such, we aimed to assess whether polyphenol intake is prospectively associated with lower depressive symptoms in young adults using longitudinal data from the Raine Study.

## Methods

### Pre-registration and ethics approval

This study was prospectively registered with the Open Science Framework registry (internet archive link: https://archive.org/details/osf-registrations-s8cwk-v1) and reported in accordance with the Strengthening the Reporting of Observational Studies in Epidemiology – Nutritional Epidemiology (STROBE-nut)^(30)^ (online Supplementary Table S1). The University of Western Australia Human Research Ethics Committee has provided overarching ethical approval (RA/4/20/5722), recognising individual approvals for each follow-up of the Raine Study. For the present study, the Deakin University Human Research Ethics Committee granted an exemption from ethical review in accordance with the National Statement on Ethical Conduct in Human Research (2007, updated 2018; project number: 2023-105).

### Cohort profile

A detailed description of the Raine Study’s cohort profile, data collection, recruitment and enrolment procedures and loss to attrition data has been described elsewhere^(31,32)^. Briefly, the Raine Study is an ongoing multigenerational study that recruited 2730 pregnant women at 16–20 weeks’ gestation visiting King Edward Memorial Hospital in Perth, Western Australia between 1989 and 1991 (the Raine Study Generation 1 participants). The offspring of the Raine Study Generation 1 participants (the Raine Study Generation 2 participants (Gen2); *n* 2868) were born between 1989 and 1992 and have currently been followed up seventeen times. Each follow-up included questionnaires, validated assessment tools, physical assessments and/or biological samples to assess behavioural, environmental, social and phenotypic data. The present study uses data collected at the Raine Study Generation 2–20, −22 and −27 year follow-up (Gen2–20 (2010–2012; *n* 1574), Gen2–22 (2012–2014; *n* 1236) and Gen2–27 (2016–2018; *n* 1190), respectively)). These time points were selected as the Gen2 participants were in young adulthood, and the relevant data for this study were collected. Participants were eligible for inclusion in this study if they had completed dietary and mental health questionnaires at any of the three time points. Our analyses included 964 participants from Gen2–20, 979 participants from Gen2–22 and 1094 participants from Gen2–27. Participants with missing dietary data (Gen2–20: *n* 591, Gen2–22: *n* 233, Gen2–27: *n* 54) and mental health data (Gen2–20: *n* 356, Gen2–22: *n* 144, Gen2–27: *n* 55) were excluded from the analysis. In accordance with the Raine Study Researcher Engagement Policy, data from the present study cannot be made publicly available. Access to these data requires approval from the Raine Study Scientific Management Committee.

### Exposure: polyphenol intake

**Figure 1.**
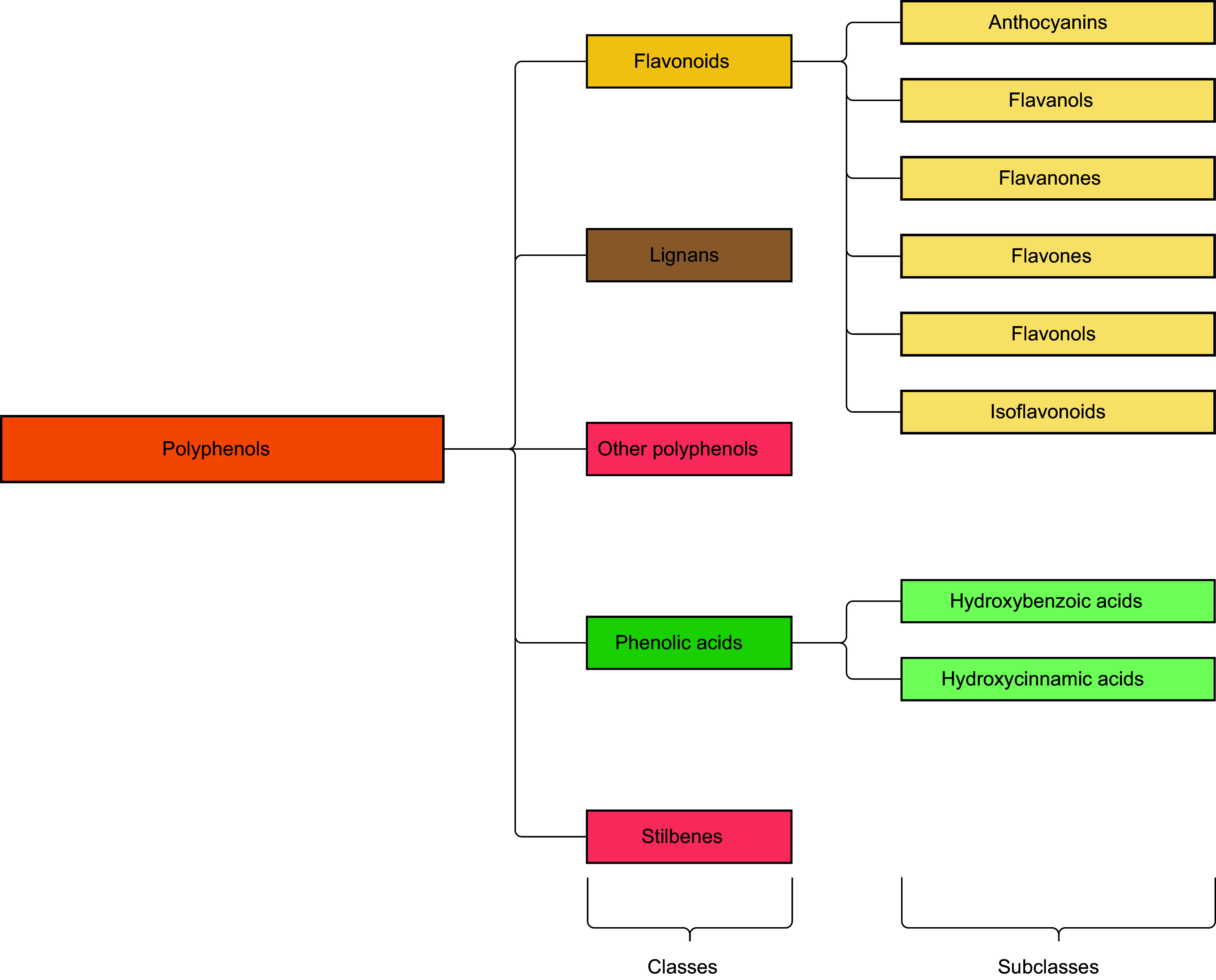
Figure 1 long description. Classification of polyphenol classes and subclasses.

Whole-of-diet habitual intakes, not including supplements, of total polyphenols, polyphenol classes and polyphenol subclasses (Figure 1) were assessed via secondary analysis from FFQ (Dietary Questionnaire for Epidemiological Studies version 2 (DQESv2)^(33)^ at Gen2–20 and Gen2–22; Dietary Questionnaire for Epidemiological Studies version 3.2 (DQESv3.2)^(34)^ at Gen2–27). Briefly, the DQESv2 (seventy-four items) and DQESv3.2 (eighty items) have been validated to assess habitual dietary intake in adults, and the DQESv2 has been validated in young adults^(35–37)^. Both questionnaires require respondents to report their average consumption of specific food and beverage items over the previous 12 months^(33,34)^. Additionally, as the DQESv2 does not include frequency questions regarding tea and coffee consumption, two large sources of polyphenols^(38)^, total polyphenols, polyphenol classes and polyphenol subclasses were also assessed via secondary analysis from self-reported frequency of cups of tea and coffee consumed at Gen2–20 and Gen2–22. Specifically, at Gen2–20 and Gen2–22, participants completed a questionnaire in which they self-reported the frequency of cups of tea, herbal tea, green tea, instant coffee and ground coffee via twelve response options ranging from ‘never’ to ‘everyday’. Cups of tea and coffee consumed per d were then converted to millilitres per d by multiplying cups by 250 ml.

Polyphenols were assigned to dietary data using our previous expansion of the 2011–13 Australian Food, Supplement and Nutrient Database (AUSNUT 2011–13) to include polyphenol content data, as described previously^(39)^, and the Phenol-Explorer database corrected version of 3.6^(40)^. First, the DQESv2 and DQESv3.2 food items that could be directly matched with Phenol-Explorer (e.g. bananas) were matched using similar methods to our previous expansion of the AUSNUT 2011-13^(39)^. For all other food items including more complex foods (e.g. pizza), we assigned polyphenols using our previous expansion of the AUSNUT 2011–13^(39)^. If the literature were available, polyphenol composition was calculated with consideration of consumption patterns; otherwise, similar to Huang *et al.* ^(38)^, polyphenol composition was calculated as the average of each relevant food item. For example, the food item ‘*Rice*’ was calculated based on the consumption ratio of 90 % white rice and 10 % brown rice^(41)^. The DQESv2 and DQESv3.2 with assigned polyphenol content data are included in the supplementary files (online Supplementary Tables S2 and S3). Similarly, regarding Gen2–20 and Gen2–22 tea and coffee intake, the questionnaire items ‘tea’ and ‘green tea’ were directly matched to the Phenol-Explorer items ‘Tea (Black), infusion’ and ‘Tea (Green), infusion’, respectively, while ‘herbal tea’ was directly matched to the AUSNUT 2011–2013 food item ‘Tea, herbal, other, without milk’. Further, due to a lack of data regarding the polyphenol content of instant and ground coffee, all coffee items were directly matched to the Phenol-Explorer item ‘Coffee beverage (Filter)’.

The weight per d (mg/d) of total polyphenols, polyphenol classes and polyphenol subclasses from each food item was then calculated by multiplying the content of each polyphenol category by the daily consumption of each food item. The weight per d (mg/d) of consumed total polyphenols, polyphenol classes and polyphenol subclasses was adjusted for energy using Willett’s residual method^(42)^ and used to model polyphenol intake. Willett’s residual method accounts for energy dilution. Therefore, this method captures the relative intake of polyphenols from foods, including those with low or negligible energy (e.g. black coffee and tea). To facilitate interpretability, polyphenol intakes not adjusted for energy intake were reported within quartiles defined by energy-adjusted intake using Willett’s residual method. Participants were categorised into quartiles according to their energy-adjusted polyphenol intake at each time point.

### Outcome: depressive symptoms

Depressive symptoms were measured using the twenty-one-item Depression, Anxiety and Stress Scale (DASS) at all three time points (Gen2–20, Gen2–22 and Gen2–27). The twenty-one-item DASS is a shortened version of the forty-two-item DASS^(43)^ and has been validated in Australian populations^(44)^, as well as young adult populations outside of Australia^(45–47)^. The twenty-one-item DASS can separately measure depression, anxiety and stress symptoms using three corresponding subscales^(48)^. Symptoms of depression, anxiety and stress are assessed in the seven days prior to using a 4-point scale for each item, ranging from 0 (‘did not apply to me at all’) to 3 (‘applied to me very much or most of the time’). Scores for each subscale can range from 0 to 21, with higher scores indicating greater depression, anxiety and/or stress^(49)^. As per the scoring instructions, twenty-one-item DASS scores were multiplied by two to align with the forty-two-item DASS^(49)^. The DASS depression subscale score (continuous) was used as our outcome variable in our main analysis.

### Potential confounders

Confounders were identified *a priori* based on previous literature^(50–52)^. Confounders were self-reported and measured at baseline (Gen2–20) and all follow-ups (Gen2–22 and Gen2–27) via self-completed surveys covering sociodemographic characteristics and lifestyle- and health-related factors. Specifically, the sociodemographic variables of interest included sex (male or female), age (continuous), education (secondary or lower, university degree or other) and employment status (employed or unemployed). Lifestyle- and health-related factors included physical activity (measured via the International Physical Activity Questionnaire^(53)^ and expressed as physical activity equivalents^(54)^ (continuous)), alcohol intake (standard drinks consumed per d; continuous) and smoking status (smoker or non-smoker). Further adjustment for nutrient content was not conducted due to a high probability of collinearity (e.g. vitamin C and flavanones).

### Statistical analysis

Characteristics of participants in different quartiles of energy-adjusted total polyphenol intake were summarised by either the mean and sd or the median and interquartile range for continuous variables and frequency and percentage for categorical variables.

To evaluate the association between quartiles of polyphenol intake categories and depressive symptoms averaged across the three time points (Gen2–20, Gen2–22 and Gen2–27), we fitted two linear mixed-effects models: model 1, unadjusted model, and model 2, adjusted for sex and time-updated confounders including age, education, employment status, physical activity, alcohol intake and smoking status. In both models, the exposure variables (intakes of total polyphenols, polyphenol classes and polyphenol subclasses) were included as time-updating exposures (seven years), and nominal measurement time points were included as fixed effects to account for the repeated measure of the design. The within-participants autocorrelation was accounted for by using an exchangeable covariance structure. The magnitudes of association across quartiles of polyphenol intake were expressed as mean differences with accompanying 95 % confidence intervals (95 % CI), and the *P* for the overall difference was reported. We tested for interactions between polyphenol intake and time to assess whether associations varied across follow-up periods; no notable differences in associations were detected. Where the *P* for overall difference was < 0·05, the Q4 *v.* Q1 results were presented in the text.

To account for potential inflation of type I error rates resulting from multiple comparisons, the Simes method was used to adjust the *P* for overall difference value^(55)^ and run separately for model 1 and model 2. However, while controlling for false positives is important in confirmatory research, strict adjustment for multiple comparisons is less critical in exploratory studies^(56,57)^. Consequently, given the exploratory nature of our aim, the main text reports the results before multiple comparisons testing. Data analysis was carried out using Stata 16.1 and Stata 18.0 software (StataCorp).

### Sensitivity analyses

Considering the small size, rather than excluding baseline participants with a DASS depression subscale cut-off score of ≥ 14^(49)^ and a baseline self-reported diagnosis of depression, these participants were excluded as two separate sensitivity analyses. An additional sensitivity analysis was conducted to reduce the possibility of sampling biases by excluding baseline participants with a self-reported diagnosis of anxiety. Rather than including BMI as a confounding variable, obesity, as defined by a BMI of ≥ 30 kg/m^2^, was considered a mediating variable. As such, we ran a sensitivity analysis excluding baseline participants with a BMI of ≥ 30 kg/m^2^. Further, to explore if energy adjustment influenced the associations, we conducted sensitivity analyses using quartiles of polyphenol intakes not adjusted for energy intake. Additional sensitivity analyses for additional health conditions were not conducted due to limited cases (e.g. three participants self-reported a diabetes diagnosis at baseline).

### Subgroup analyses

Considering the high rate of missing data for lifestyle factors, subgroup analyses were conducted for each lifestyle factor to investigate the model-based estimated association between polyphenol intake and depressive symptoms in each lifestyle stratum. This approach mitigates the risk of selection bias from multiple imputation techniques that assume a missing at random pattern for missingness^(58)^. Additionally, given the protective role for lifestyle factors including physical activity^(59)^, non-smoking^(60)^ and low alcohol consumption^(61)^ against depressive outcomes, we conducted subgroup analyses to minimise the influence of these external factors. Participants remained in the same quartiles of polyphenol intake as our main analysis but were further categorised according to baseline lifestyle risks: current smoking status (yes/no), physical activity measured by the International Physical Activity Questionnaire^(53)^ and according to the WHO and Australian physical activity guidelines^(54,62)^ (met physical activity guidelines, did not meet physical activity guidelines) and standard drinks consumed per d (nil to low (< 2), moderate to high (≥ 2)^(63)^). Additionally, to assess sex specific differences in associations, participants were categorised according to their sex (male, female).

### Exploratory analyses

Three exploratory analyses were conducted. First, to examine the association between polyphenol intake and the incidence of depression, participants were categorised as depressed or non-depressed based on the DASS depression subscale cut-off score of ≥ 14 at baseline^(49)^. Second, to explore the association between polyphenol intake and overall mental health symptoms, the total DASS score averaged across time was used as the outcome. Third, considering (a) alcoholic beverages are large sources of polyphenols^(38)^ and (b) the possible harmful association between alcohol intake and depression in young adults^(64)^, polyphenols from alcohol were excluded from the analysis.

## Results

### Participant characteristics

The study comprised 1484 participants in total (52·6% female), with 964 included at Gen2–20, 979 at Gen2–22 and 1094 at Gen2–27, with overlap across follow-ups. Of the total sample, 522 participants (35·2 %) contributed data at all three time points, 509 (34·3 %) at two and 453 (30·5 %) at one.

Sociodemographic and lifestyle characteristics at baseline (Gen2–20) by quartiles of energy-adjusted total polyphenol intake are presented in Table 1. Compared with the lowest quartile of total polyphenol intake, the highest quartile appeared to have more females, current smokers, employed participants and participants whose highest education achieved was a university degree. Further, compared with participants in the lowest quartile, participants in the highest quartile appeared to exercise less and have a lower BMI. Participants in the lowest and highest quartiles appeared to be of similar age and consumed a similar amount of energy and standard drinks per d. Food items contributing to total polyphenols and polyphenol classes at baseline are shown in Table 2. The top contributors to total polyphenols and polyphenol classes at Gen2–22 and Gen2–27 and the top contributors to polyphenol subclasses at Gen2–20, Gen2–22 and Gen2–27 are included as supplementary materials (online Supplementary Tables S4–S8).

**Table 1.** Baseline participant characteristics according to quartiles of polyphenol intake after adjusting for energy Table 1 long description.

	Quartile 1		Quartile 2		Quartile 3		Quartile 4		Combined	
	n	% female	n	% female	n	% female	n	% female	n	% female
No. of participants	241	40·2%	241	58·1%	241	58·9%	241	53·1%	964	52·6%
	Median	Range	Median	Range	Median	Range	Median	Range	Median	Range
Total polyphenol intake										
Energy adjusted (mg/day)^*^	−498·97	−1271·92, −411·82	−322·77	−410·42, −199·96	−27·68	−198·51, 256·59	683·98	257·95, 8127·37	−199·23	−1271·92, 8127·37
Energy unadjusted (mg/day)^†^	262·72	57·02, 1525·01	429·29	147·68, 1286·03	763·75	398·24, 1546·59	1498·94	767·04, 8894·07	583·17	57·02, 8894·07

	Mean	SD	Mean	SD	Mean	SD	Mean	SD	Mean	SD
Energy intake (kJ)	9132·6	5382·7	7558·8	3319·2	8350·5	3998·6	9012·7	4667·1	8513·6	4446·6
Age (years)	20	0·5	20	0·4	20	0·5	20	0·5	20	0·5
BMI	25·2	6·1	24·5	4·9	24·0	4·8	24·1	4·9	24·4	5·2
	n	%	n	%	n	%	n	%	n	%
Education^‡^										
Secondary or lower	38	36·2%	28	30·4%	25	28·1%	29	29·6%	120	31·3%
University degree	11	10·5%	18	19·6%	16	18·0%	21	21·4%	66	17·2%
Other	56	53·3%	46	50·0%	48	53·9%	48	49·0%	198	51·6%
Employment status										
Unemployed	46	19·1%	45	18·7%	47	19·5%	34	14·1%	172	17·8%
Employed	195	80·9%	196	81·3%	194	80·5%	207	85·9%	792	82·2%

	Mean	SD	Mean	SD	Mean	SD	Mean	SD	Mean	SD
Physical activity^§^										
Vigorous activity (mins/day)	63	69	51	57	55	60	55	60	56	62
Moderate activity (mins/day)	54	69	46	57	52	62	44	60	49	62
	n	%	n	%	n	%	n	%	n	%
Current smoker ^||^										
No	205	85·1%	218	90·8%	218	90·5%	189	78·4%	830	86·2%
Yes	36	14·9%	22	9·2%	23	9·5%	52	21·6%	133	13·8%

	Mean	SD	Mean	SD	Mean	SD	Mean	SD	Mean	SD
Standard drinks (per day)	1·9	2·1	1·4	1·7	1·7	1·9	1·9	2·1	1·7	2·0
DASS score										
Total score	20·5	19·1	20·7	19·6	20·3	18·4	22·8	20·4	21·1	19·4
Depression subscale	7·1	7·9	7·2	8·5	6·6	7·4	7·2	8·1	7·0	8·0
Anxiety subscale	5·0	5·5	5·1	5·5	4·8	5·5	5·5	6·3	5·1	5·7
Stress subscale	8·4	8·1	8·4	7·9	8·9	8·0	10·0	8·3	8·9	8·1

DASS, Depression, Anxiety and Stress Scale.

* Energy-adjusted values derived using Willett’s residual method; negative values indicate intake below that predicted for total energy intake.

† Energy-unadjusted values reflect intakes within energy-adjusted polyphenol intake quartiles.

‡ Education data were missing at baseline for 580 participants.

§ Physical activity data were missing at baseline for three participants.

|| Current smoking status data were missing at baseline for one participant.

**Table 2.** The three highest food item contributions to total polyphenols, and polyphenol classes at baselin Table 2 long description.

Polyphenol class	Top three contributors	Percentage contribution
Total polyphenols	Coffee	37·7%
	Black tea	13·6%
	Apples	8·0%
Flavonoids	Black tea	24·2%
	Apples	15·5%
	Chocolate	10·5%
Lignans	Bread – multigrain	68·3%
	Broccoli	3·6%
	Muesli	3·6%
Other polyphenols	Sultana Bran, Fibre Plus, Bran Flakes	16·3%
	Weet Bix, Vita Brits, Weeties	14·9%
	Bread – wholemeal	12·5%
Phenolic Acids	Coffee	72·9%
	Black tea	5·6%
	Apples	2·1%
Stilbenes	Red wine	64·2%
	White wine	28·9%
	Strawberries	4·0%

**Table 3.** Mean differences and 95% confidence intervals for the prospective association between energy-adjusted intake of total polyphenols and polyphenol classes and DASS depression subscale scores Table 3 long description.

Polyphenol class	Quartile 1	Quartile 2	Quartile 3	Quartile 4	P for overall difference	Q value^*^
**Total polyphenols**						
Energy-adjusted (mg/day)^†^ Median (IQR)	−581·15 (−692·09, −516·53)	−322·67 (−387·94, −247·84)	26·14 (−78·34, 186·88)	696·53 (525·83, 1041·15)		
Energy-unadjusted (mg/day)^‡^ Median (IQR)	306·75 (218·16, 441·47)	564·40 (439·82, 740·83)	962·05 (780·89, 1177·00)	1717·91 (1440·42, 2046·22)		
Model 1	Referent	−0·37 (−1·14, 0·39)	−0·58 (−1·38, 0·22)	−0·47 (−1·30, 0·36)	0·54	0·89
Model 2	Referent	−0·59 (−1·67, 0·49)	−0·10 (−1·21, 1·00)	−0·33 (−1·43, 0·78)	0·71	0·87
**Flavonoids**						
Energy-adjusted (mg/day)^†^ Median (IQR)	−216·72 (−256·01, −190·97)	−115·23 (−140·66, −93·00)	2·82 (−34·21, 45·68)	259·57 (167·42, 418·05)		
Energy-unadjusted (mg/day)^‡^ Median (IQR)	150·67 (113·52, 197·87)	242·21 (199·10, 292·39)	372·90 (320·59, 434·76)	650·10 (548·23, 824·08)		
Model 1	Referent	−0·01 (−0·76, 0·75)	0·28 (−0·50, 1·06)	−0·05 (−0·87, 0·76)	0·80	0·89
Model 2	Referent	−0·14 (−1·21, 0·93)	−0·21 (−1·29, 0·86)	−0·41 (−1·52, 0·70)	0·91	0·91
**Lignans**						
Energy-adjusted (mg/day)^†^ Median (IQR)	−2·75 (−3·12, −2·51)	−2·05 (−2·21, −1·80)	−1·10 (−1·60, −0·33)	5·08 (2·02, 7·99)		
Energy-unadjusted (mg/day)^‡^ Median (IQR)	0·80 (0·57, 1·15)	0·84 (0·61, 1·22)	1·74 (1·09, 2·53)	8·25 (5·15, 11·79)		
Model 1	Referent	0·34 (−0·42, 1·10)	−0·33 (−1·11, 0·45)	0·06 (−0·72, 0·84)	0·37	0·89
Model 2	Referent	0·26 (−0·84, 1·37)	−0·31 (−1·40, 0·79)	−0·11 (−1·18, 0·96)	0·77	0·87
**Other polyphenols**						
Energy-adjusted (mg/day)^†^ Median (IQR)	−20·96 (−26·04, −17·29)	−9·52 (−12·02, −7·08)	0·72 (−2·28, 4·05)	21·14 (13·68, 37·84)		
Energy-unadjusted (mg/day)^‡^ Median (IQR)	11·69 (7·45, 17·68)	20·00 (13·97, 26·94)	31·83 (25·00, 37·86)	55·77 (45·80, 73·98)		
Model 1	Referent	−0·19 (−0·95, 0·57)	−0·07 (−0·85, 0·70)	−0·38 (−1·17, 0·42)	0·79	0·89
Model 2	Referent	0·46 (−0·63, 1·54)	−0·17 (−1·25, 0·90)	0·34 (−0·75, 1·43)	0·58	0·87
**Phenolic acids**						
Energy-adjusted (mg/day)^†^ Median (IQR)	−432·32 (−504·08, −344·54)	−278·90 (−339·89, −242·78)	−44·29 (−110·60, 44·51)	632·71 (434·50, 777·66)		
Energy-unadjusted (mg/day)^‡^ Median (IQR)	81·48 (53·82, 123·47)	176·01 (110·55, 309·14)	503·13 (330·39, 598·99)	1238·96 (869·76, 1364·40)		
Model 1	Referent	−0·38 (−1·14, 0·38)	−0·64 (−1·43, 0·15)	−0·61 (−1·44, 0·21)	0·40	0·89
Model 2	Referent	−0·68 (−1·77, 0·40)	−0·11 (−1·21, 0·98)	−0·33 (−1·43, 0·77)	0·61	0·87
**Stilbenes**						
Energy-adjusted (mg/day)^†^ Median (IQR)	−1·07 (−1·17, −1·01)	−0·86 (−0·93, −0·73)	−0·44 (−0·62, −0·24)	1·43 (0·59, 3·06)		
Energy-unadjusted (mg/day)^‡^ Median (IQR)	0·06 (0·03, 0·10)	0·11 (0·05, 0·20)	0·54 (0·35, 0·76)	2·52 (1·66, 4·19)		
Model 1	Referent	0·43 (−0·34, 1·20)	0·57 (−0·22, 1·36)	0·73 (−0·11, 1·56)	0·35	0·89
Model 2	Referent	0·59 (−0·54, 1·72)	0·22 (−0·87, 1·32)	0·28 (−0·91, 1·47)	0·78	0·87

DASS, Depression, Anxiety and Stress Scale; IQR, interquartile range.

Model 1. Unadjusted for any covariate (*n* 1484).

Model 2. Adjusted for age, sex, education, employment, physical activity, smoking status and alcohol intake (*n* 1137); reduced sample size due to missing covariate data.

* Adjusted *P* for overall difference using the Simes method.

† Energy-adjusted values derived using Willett’s residual method; negative values indicate intake below that predicted for total energy intake.

‡ Energy-unadjusted values reflect intakes within energy-adjusted polyphenol intake quartiles.

**Table 4.** Mean differences and 95% confidence intervals for the prospective association between energy-adjusted intake of polyphenol subclasses and DASS depression subscale scores Table 4 long description.

Polyphenol subclass	Quartile 1	Quartile 2	Quartile 3	Quartile 4	P for overall difference	Q value^*^
**Anthocyanins**						
Energy-adjusted (mg/day)^†^ Median (IQR)	−17·70 (−21·47, −14·96)	−9·29 (−11·39, −7·47)	−1·29 (−3·67, 2·03)	20·94 (12·14, 37·39)		
Energy-unadjusted (mg/day)^‡^ Median (IQR)	6·23 (3·47, 9·42)	11·71 (7·78, 15·81)	20·55 (15·42, 26·75)	45·55 (34·64, 63·08)		
Model 1	Referent	−0·25 (−1·00, 0·51)	−0·35 (−1·12, 0·41)	−0·41 (−1·20, 0·39)	0·76	0·89
Model 2	Referent	−0·39 (−1·47, 0·68)	−0·54 (−1·61, 0·54)	−0·44 (−1·53, 0·64)	0·78	0·87
**Flavanols**						
Energy-adjusted (mg/day)^†^ Median (IQR)	−171·24 (−202·63, −153·44)	−98·33 (−117·40, −78·78)	−3·79 (−34·06, 30·12)	217·32 (134·45, 341·57)		
Energy-unadjusted (mg/day)^‡^ Median (IQR)	83·75 (59·27, 117·07)	155·37 (124·39, 183·93)	257·01 (221·44, 301·99)	485·68 (403·97, 628·73)		
Model 1	Referent	0·01 (−0·74, 0·77)	0·23 (−0·54, 1·01)	0·20 (−0·61, 1·02)	0·91	0·91
Model 2	Referent	−0·59 (−1·65, 0·48)	−0·30 (−1·37, 0·77)	−0·51 (−1·63, 0·60)	0·71	0·87
**Flavanones**						
Energy-adjusted (mg/day)^†^ Median (IQR)	−21·15 (−26·00, −17·36)	−11·81 (−14·72, −9·80)	−1·25 (−4·50, 3·45)	24·94 (15·39, 43·55)		
Energy-unadjusted (mg/day)^‡^ Median (IQR)	6·01 (2·78, 10·48)	11·59 (7·24, 17·86)	24·78 (17·37, 33·39)	56·34 (41·18, 76·46)		
Model 1	Referent	0·36 (−0·37, 1·10)	0·09 (−0·67, 0·84)	−0·09 (−0·85, 0·68)	0·67	0·89
Model 2	Referent	0·72 (−0·31, 1·76)	0·79 (−0·26, 1·84)	0·65 (−0·40, 1·69)	0·43	0·87
**Flavones**						
Energy-adjusted (mg/day)^†^ Median (IQR)	−7·80 (−10·30, −6·33)	−3·94 (−4·71, −3·23)	−0·60 (−1·63, 0·65)	9·65 (5·92, 17·28)		
Energy-unadjusted (mg/day)^‡^ Median (IQR)	4·25 (2·71, 6·83)	4·85 (3·24, 6·62)	7·61 (5·48, 10·11)	20·82 (14·94, 31·35)		
Model 1	Referent	0·27 (−0·46, 1·01)	0·07 (−0·68, 0·82)	−0·06 (−0·83, 0·70)	0·83	0·89
Model 2	Referent	0·23 (−0·82, 1·28)	0·43 (−0·64, 1·49)	−0·04 (−1·11, 1·04)	0·81	0·87
**Flavonols**						
Energy-adjusted (mg/day)^†^ Median (IQR)	−24·06 (−29·16, −20·45)	−14·02 (−16·41, −11·54)	−1·14 (−5·32, 3·90)	30·50 (17·91, 51·13)		
Energy-unadjusted (mg/day)^‡^ Median (IQR)	14·87 (11·61, 19·64)	25·23 (19·96, 32·23)	40·11 (31·66, 48·88)	74·27 (58·80, 94·68)		
Model 1	Referent	−0·75 (−1·50, 0·00)	−0·40 (−1·18, 0·38)	−0·65 (−1·46, 0·16)	0·22	0·89
Model 2	Referent	−1·58 (−2·64, −0·53)	−0·96 (−2·05, 0·13)	−1·38 (−2·48, −0·28)	0·02	0·09
**Isoflavonoids**						
Energy-adjusted (mg/day)^†^ Median (IQR)	−6·40 (−6·94, −4·15)	−5·77 (−6·17, −3·87)	−2·64 (−3·83, −1·57)	5·80 (1·67, 14·18)		
Energy-unadjusted (mg/day)^‡^ Median (IQR)	0·03 (0·01, 0·05)	0·08 (0·04, 0·34)	2·41 (1·66, 3·87)	11·44 (7·02, 19·92)		
Model 1	Referent	−0·70 (−1·43, 0·03)	−0·69 (−1·44, 0·06)	0·37 (−0·41, 1·14)	0·008	0·11
Model 2	Referent	−1·52 (−2·57, −0·47)	−1·26 (−2·31, −0·21)	−0·29 (−1·37, 0·79)	0·009	0·08
**Hydroxybenzoic acids**						
Energy-adjusted (mg/day)^†^ Median (IQR)	−17·84 (−20·02, −14·85)	−11·67 (−14·12, −8·97)	−2·71 (−5·82, 0·77)	20·73 (11·03, 38·95)		
Energy-unadjusted (mg/day)^‡^ Median (IQR)	6·61 (4·43, 9·12)	15·92 (12·00, 19·17)	31·85 (25·31, 39·63)	80·48 (52·62, 102·47)		
Model 1	Referent	−0·40 (−1·16, 0·36)	−0·18 (−0·97, 0·60)	−0·36 (−1·18, 0·47)	0·73	0·89
Model 2	Referent	−1·58 (−2·67, −0·49)	−1·69 (−2·80, −0·59)	−1·40 (−2·53, −0·27)	0·01	0·08
**Hydroxycinnamic acids**						
Energy-adjusted (mg/day)^†^ Median (IQR)	−249·72 (−283·51, −197·58)	−152·92 (−182·37, −128·43)	−29·39 (−66·22, 21·19)	312·50 (207·21, 499·35)		
Energy-unadjusted (mg/day)^‡^ Median (IQR)	57·77 (40·05, 74·66)	148·08 (99·62, 286·73)	475·16 (282·36, 574·80)	1209·79 (822·45, 1299·08)		
Model 1	Referent	−0·94 (−1·69, −0·19)	−0·90 (−1·70, −0·11)	−0·59 (−1·41, 0·24)	0·06	0·43
Model 2	Referent	−0·83 (−1·92, 0·27)	−0·56 (−1·68, 0·55)	−0·19 (−1·32, 0·94)	0·43	0·87

DASS, Depression, Anxiety and Stress Scale; IQR, interquartile range.

Model 1. Unadjusted for any covariate (*n* 1484).

Model 2. Adjusted for age, sex, education, employment, physical activity, smoking status and alcohol intake (*n* 1137); reduced sample size due to missing covariate data.

* Adjusted *P* for overall difference using the Simes method.

† Energy-adjusted values derived using Willett’s residual method; negative values indicate intake below that predicted for total energy intake.

‡ Energy-unadjusted values reflect intakes within energy-adjusted polyphenol intake quartiles.

### Association between total polyphenols and polyphenol classes and depressive symptoms

In our unadjusted model (model 1) and multivariable model (model 2), we observed little to no evidence of an association with depressive symptoms averaged across time for total polyphenols and all polyphenol classes (Table 3).

For total polyphenols and all polyphenol classes, the magnitude and direction of association were mostly consistent with our main multivariable model (model 2) following sensitivity analyses (excluding baseline participants with a DASS depression subscale cut-off score of ≥ 14, a self-reported diagnosis of depression, a self-reported diagnosis of anxiety and a BMI of ≥ 30 kg/m^2^ and using quartiles of energy-unadjusted polyphenol intakes (online Supplementary Tables S9–S13)) and exploratory analyses (depression as a binary outcome, DASS total score as the outcome and after excluding polyphenols from alcohol (online Supplementary Tables S14–S16)). Following lifestyle factor subgroup analyses (current smoking status at baseline (yes/no), met physical activity guidelines at baseline (yes/no) and standard drinks consumed per d at baseline (nil to low/moderate to high)), the magnitude and direction of association for total polyphenols and all polyphenol classes were somewhat consistent (online Supplementary Tables S17 and S19). Following sex subgroup analyses, the direction of association for phenolic acids was consistent with our main multivariable model (model 2) in both males and females; however, the magnitude was larger in females. Among females only, the direction of associations was consistent for total polyphenols, flavonoids and lignans, with a greater magnitude compared with our main multivariable model (online Supplementary Table S20).

### Association between polyphenol subclasses and depressive symptoms

In our unadjusted model (model 1), we observed little to no evidence of an association with depressive symptoms averaged across time for all polyphenol subclasses except for isoflavonoids, where we observed strong evidence of an association (*P* for overall difference = 0·008) (Table 4). However, when comparing participants with the lowest intake of isoflavonoids to participants in the highest, no association was observed (Q4 *v.* Q1 mean difference: 0·37; 95 % CI −0·41, 1·14).

In our multivariable model (model 2), we observed strong evidence of an association with depressive symptoms averaged across time for isoflavonoid intake (*P* for overall difference = 0·009) and moderate evidence of an association for flavonol (*P* for overall difference = 0·02) and hydroxybenzoic acid intake (*P* for overall difference = 0·01) (Table 4). When comparing participants with the lowest intake of flavonol and hydroxybenzoic acid intake, participants with the highest intake reported lower depressive symptoms (flavonols (Q4 *v.* Q1 mean difference: −1·38; 95% CI −2·48, −0·28); hydroxybenzoic acids (Q4 *v.* Q1 mean difference: −1·40; 95 % CI −2·53, −0·27)). However, little to no evidence of an association was observed when comparing participants with the highest intake of isoflavonoids to those with the lowest (Q4 *v.* Q1 mean difference: −0·29; 95 % CI −1·37, 0·79). Little to no evidence of an association was observed across quartiles for all other polyphenol subclasses in our multivariable model (model 2).

Regarding flavonols and hydroxybenzoic acids, the magnitude and direction of associations with depressive symptoms in our main multivariable model (model 2) were consistent with associations observed in our sensitivity analyses (excluding baseline participants with a DASS depression subscale cut-off score of ≥ 14, a self-reported diagnosis of depression, a self-reported diagnosis of anxiety and a BMI of ≥ 30 kg/m^2^ and using quartiles of energy-unadjusted polyphenol intakes (online Supplementary Tables S21–S25)) and our exploratory analyses (depression as a binary outcome, DASS total score as the outcome and after excluding polyphenols from alcohol (online Supplementary Tables S26–S28)). Following subgroup lifestyle factor analyses, the associations for flavonols and hydroxybenzoic acids were consistent with our multivariable model (model 2) regardless of physical activity guideline adherence, in nil to low alcohol consumers and non-smokers (online Supplementary Tables S29–S31). Following sex subgroup analyses, the direction of association for flavonols and hydroxybenzoic acids was similar to that observed in the multivariable model (model 2) in both males and females; however, the magnitude was greater in females (online Supplementary Table S32).

## Discussion

To our knowledge, this is the first study to assess the prospective association between dietary polyphenol intake and depressive symptoms in young adults. In our cohort, higher intakes of flavonols and hydroxybenzoic acids were associated with lower depressive symptoms.

### Comparison with previous studies

Several cross-sectional studies have assessed the association between polyphenol intake and depressive outcomes^(22,23,65–69)^. While many of the studies reported inverse associations between the intakes of total flavonoids^(22,66,67)^ and several flavonoid subclasses and depressive outcomes, including anthocyanins^(22,23,65,69)^, flavanones^(22,23,66,67)^, flavones^(22,65–67)^ and isoflavones^(22)^, similar to the present study, one study reported an inverse association for flavonols^(66)^. Additionally, of the two studies that assessed the association in non-flavonoids, phenolic acids were the only other class of polyphenols reported to be associated with depressive outcomes, with one study reporting an inverse association^(23)^ and the other reporting a direct association^(68)^.

While cross-sectional studies offer valuable snapshots, prospective studies support temporal inferences; two such studies have previously been published assessing the association between the intake of a variety of polyphenols and depressive outcomes^(24,25)^. Bardinet *et al*.’s^(25)^ study assessed the association between patterns of polyphenol intake and risk of depressive symptomatology over 16 years in older adults (*n* 1074; ≥ 65 years). Their study reported a lower risk in participants with greater adherence to a pattern primarily shaped by monomeric flavanols and theaflavins (OR = 0·73; 95 % CI 0·55, 0·97). Chang *et al*.’s^(24)^ study assessed the association between flavonoid intake and depression risk. Similar to the present study, in their pooled analysis, participants with higher intakes of flavonols had a lower risk of depression (hazard ratio = 0·93; 95 % CI 0·88, 0·99), while no association was reported for flavonoid, anthocyanin and flavanol intake^(24)^. However, unlike the present study, an association was reported for flavone and flavanone intake (hazard ratio = 0·92; 95 % CI 0·86, 0·98 and hazard ratio = 0·90; 95 % CI 0·85, 0·96, respectively)^(24)^. Although the results from our study and those from Chang e*t al*.^(24)^ differed, this could be due to differences in study design. For example, in Chang *et al*.’s^(24)^ study, their participants were older (age range: 36–80 years) and all female, their sample size was larger (*n* 82 643) and their follow-up time was longer (10 years). However, the difference in results may also suggest that associations between flavone and flavanone intake and risk of depression may be less evident earlier in the lifespan or that longer periods are required for associations to become evident. As such, future studies are required to assess the association between polyphenols and depression across the lifespan and over longer study durations.

Beyond observational research, intervention studies have assessed the effects of flavonol and hydroxybenzoic acid supplementation on depressive symptoms^(70,71)^. For example, in a recent randomised controlled trial, individuals with diagnosed major depressive disorder were randomised to receive either 200 mg of ellagic acid (a hydroxybenzoic acid; intervention) or matched placebo (control) daily for 8 weeks. The study reported reduced depressive symptoms in the intervention group compared with the control group^(70)^. An additional 8-week randomised controlled trial assessed the effect of 500 mg of the flavonol quercetin on depressive symptoms in individuals who had experienced a myocardial infarction^(71)^. The study reported reduced depressive symptoms compared with baseline in the intervention group; however, no difference was reported compared with controls^(71)^. Notably, the doses of ellagic acid and quercetin were higher than the daily median quartile 4 intakes of total hydroxybenzoic acids and flavonols observed in the present study (80·48 mg and 74·27 mg, respectively). Further, considering the multitude of individual polyphenol compounds within the flavonol and hydroxybenzoic acid subclasses, future intervention studies should assess the effects of other flavonol and hydroxybenzoic acid polyphenols.

### Potential mechanisms

While observational and interventional studies collectively suggest potential benefits, the underlying mechanisms through which polyphenols are beneficial for depression remain unclear, particularly in human models. However, animal studies suggest that polyphenols modulate pathways implicated in depression including inflammation, oxidative stress, the hypothalamic–pituitary–adrenal axis and neurogenesis^(20)^. Indeed, relevant to the results of the present study, rodent studies have reported that administration of flavonols (e.g. kaempferol and quercetin) and hydroxybenzoic acids (e.g. paeoniflorin and punicalin) mitigates depressive-like behaviours, reduces neuroinflammation and hypothalamic–pituitary–adrenal axis hyperfunction and increases concentrations of antioxidant enzymes and markers of neurogenesis^(72–77)^. Further, evidence suggests that polyphenols may be beneficial for depression due to their reciprocal relationship with the gut microbiota^(78)^. Polyphenols are poorly absorbed in the small intestine^(79)^. As such, polyphenols reach the gut microbiota where they function as prebiotics, facilitating the growth of beneficial bacteria including bacteria genera belonging to families linked to improved depressive outcomes (e.g. *Lachnospiraceae* family)^(80,81)^. In return, the gut microbiota metabolise polyphenols producing bioactive metabolites, some of which have been shown to reduce depressive-like symptoms in animal models including equol, a metabolite of daidzein^(82)^. Considering the current mechanistic research is largely limited to animal studies, future research is necessary in humans to better understand the interconnected relationships between polyphenols, the gut microbiota and the pathophysiological pathways of depression.

### Dietary sources and contextual considerations

In addition to their potential mechanistic effects, flavonols and hydroxybenzoic acids were primarily consumed from tea at all three time points in our study. Higher tea intake has specifically been associated with lower depressive outcomes in a meta-analysis of prospective and cross-sectional studies^(83)^. Although tea contains other healthful nutritive components, its high polyphenol content, combined with results from our study, suggests that it may be, at least in part, the polyphenol content of tea that confers health benefits^(84)^.

### Methodological considerations and implications for future directions

Our findings remained robust across multiple sensitivity and subgroup analyses. Excluding baseline participants with a self-reported diagnosis of depression or a DASS depression subscale score of ≥ 14 did not meaningfully change the magnitude or direction of the associations for either flavonols or hydroxybenzoic acids. This suggests these findings are robust and unlikely to be influenced by the presence of depression at baseline. Furthermore, subgroup analyses stratified by baseline lifestyle factors indicated that the associations for both flavonols and hydroxybenzoic acids persisted among non-smokers, those with nil to low alcohol consumption and irrespective of physical activity levels. However, in current smokers and moderate to high alcohol consumers, the direction of the associations was reversed, suggesting that certain lifestyle factors may influence the relationship between flavonol and hydroxybenzoic acid intake and depressive symptoms. Moreover, the associations for flavonols and hydroxybenzoic acids appeared more pronounced in females than in males. Together, these sensitivity and subgroup analyses provide additional support for our main findings while highlighting important areas for future research to explore the potential modifying effects of lifestyle and demographic variables.

As exposure and outcome data were assessed at baseline (Gen2–20) and all follow-ups (Gen2–22 and Gen2–27), our study provided a comprehensive assessment of the association between polyphenol intake and depressive symptoms. However, due to relatively small participant numbers, the findings of our study may have limited generalisability to the broader young adult population. Additionally, our study’s reliance on self-reported data for both the exposure and outcome variables introduces the potential for recall bias, where participants may inaccurately recall past events or experiences, and social desirability bias, where participants may respond in a way they perceive as socially acceptable^(85)^. However, depressive symptoms were assessed using a validated mental health instrument, which, despite being self-reported, is a standard approach in large epidemiological studies^(43,86)^.

Accurate measurement of dietary polyphenol intake presents additional methodological challenges. For example, current polyphenol food composition databases have limitations that impair the precision of current estimates, as discussed elsewhere^(87)^. Notably, the polyphenol content of food varies widely depending on geographic location^(88,89)^, farming practices (i.e. conventional *v.* organic; conventional *v.* regenerative)^(90,91)^ and plant maturity^(92)^, which are not considered in current polyphenol food composition databases. Further, while the DQESv2 and DQESv3.2 have been validated to assess habitual dietary intake in adults, and the DQESv2 has been validated in young adults^(35–37)^, they have not been validated specifically to measure polyphenol intake. This is important considering studies have reported that some FFQ overestimate the intake of various polyphenol classes and subclasses^(93–95)^, and similar issues may apply to the DQESv2 and DQESv3.2. Although future research is required to validate the DQESv2 and DQESv3.2 to measure polyphenol intake, we used several methods to improve the precision in our study that have not been incorporated in prior studies. For example, for food items listed in the DQESv2 and DQESv3.2 that could not be matched directly to Phenol-Explorer (e.g. complex foods such as pizza), these were matched to food items listed in the AUSNUT 2011–13, to which we previously expanded to include polyphenol content data using Phenol-Explorer^(39)^. For these foods, consumption patterns were considered based on the 2011–13 Australian Health Survey, divided according to the literature or averaged between appropriate food items. This approach also helped address a common limitation of FFQ, where polyphenol-rich foods (e.g. herbs) are often not directly measured or are underrepresented. Although such foods are not captured as standalone items in the DQESv2 or DQESv3.2, their inclusion as components of composite foods (e.g. herbs in salad dressing) within the expanded AUSNUT 2011–13 database allowed us to indirectly account for some of their polyphenol content. As such, this approach strengthened our study as it increased the accuracy of polyphenol intake assessment by integrating multiple data sources specific to the Australian population. Additionally, a key strength of this study is the use of Willett’s residual method to adjust polyphenol intake for total energy intake, which reduces confounding while accounting for foods that contribute low to negligible energy, such as tea and coffee^(42)^.

Similar to population polyphenol intake studies^(38,96,97)^, tea was a major source of polyphenols in our cohort. While certain fruits and vegetables, such as apples, broccoli and strawberries, were also among the top sources, the predominance of tea, reflected in its higher percentage contribution, may indicate generally low intake of a broader range of polyphenol-rich foods, particularly fruits and vegetables, which are known to be under-consumed by young Australian adults^(98)^. It is possible that threshold effects exist, where a minimum intake of these foods is necessary to observe meaningful associations, and should be investigated in future studies.

While the magnitude of effect observed for the association with flavonol and hydroxybenzoic acid intake and depressive symptoms was somewhat modest, prevention strategies that combine increased intake of these compounds with other evidence-based approaches, such as increased physical activity, may yield potential clinically meaningful benefits^(99)^. This approach leverages the combined benefits of multiple strategies, potentially achieving greater outcomes than any single strategy alone^(100)^. However, further studies are first needed to determine whether increased intake of flavonols and/or hydroxybenzoic acids prevents depression.

Additionally, future studies may aim to assess not only the association between the intake of polyphenols and depressive symptoms but also the association between the presence of polyphenols in biological fluids and depressive symptoms. Considering the interindividual variability in polyphenol metabolism influenced by factors such as gut microbiota composition^(101)^, assessing polyphenol biomarkers may provide a more comprehensive understanding of the association between polyphenol intake and depression. A recent Mendelian randomisation study exemplified this by assessing the association between hippurate (a polyphenol biomarker) and depression^(21)^. The authors reported a lower concentration of hippurate in individuals with depression, suggesting a causal pathway^(21)^.

Given the exploratory nature of our study aim, we examined fourteen outcome variables (total polyphenols (*n* 1); polyphenol classes (*n* 5); polyphenol subclasses (*n* 8)), allowing us to assess the association with depressive symptoms for a wide range of polyphenol categories. However, as adjusting for multiple comparisons inherently inflates *P* values^(102)^, our exploratory approach increased the likelihood of losing evidence for an association after adjusting. Therefore, while the adjusted *P* for overall difference values may suggest a lack of associations, it is important to consider the exploratory context as well as the consistent associations observed across our sensitivity and exploratory analyses. Future studies should adopt a more targeted approach by assessing specific polyphenol subclasses, including flavonols and hydroxybenzoic acids, to minimise the risk of type I error.

### Conclusion

We observed moderate evidence of an association with lower depressive symptoms averaged across time for higher dietary intake of flavonols and hydroxybenzoic acids in young adults. The results from the present study, in combination with previous studies in the field, suggest that polyphenol intake may be a useful target for novel prevention strategies for depression. However, further mechanistic studies in human populations, prospective studies in young adults and across the lifespan and intervention studies assessing a range of individual flavonol and hydroxybenzoic acid compounds are required.

## Supporting information

10.1017/S0007114525105886.sm001 Gamage et al. supplementary material Gamage et al. supplementary material

## Supplementary material

For supplementary material/s referred to in this article, please visit https://doi.org/10.1017/S0007114525105886.

## Figure 1 Long description

The diagram illustrates the classification of polyphenols into various classes and subclasses. The main classes include Flavonoids, Lignans, Other polyphenols, Phenolic acids, and Stilbenes. Flavonoids are further divided into Anthocyanins, Flavanols, Flavanones, Flavones, Flavonols, and Isoflavonoids. Phenolic acids are categorized into Hydroxybenzoic acids and Hydroxycinnamic acids. The diagram uses color-coded boxes to represent each class and subclass, with connecting lines indicating their hierarchical relationships.

Navigate back to Figure 1.

## Table 1 Long description

The table presents baseline participant characteristics categorized by quartiles of polyphenol intake, adjusted for energy. It includes data on the number of participants, total polyphenol intake, energy intake, age, body mass index, education level, employment status, physical activity, smoking status, standard drinks per day, and DASS scores. The table has four quartiles, each with 241 participants, and a combined total of 964 participants. Key trends include variations in polyphenol intake, energy intake, education levels, employment status, physical activity, and smoking status across quartiles.

Navigate back to Table 1.

## Table 2 Long description

The table presents the top three contributors to different polyphenol classes and their respective percentage contributions. It includes five polyphenol classes: Total polyphenols, Flavonoids, Lignans, Other polyphenols, Phenolic Acids, and Stilbenes. Each class lists the top three food items contributing to it, along with their percentage contributions. For example, coffee is the top contributor to total polyphenols with a 37 to 77 percentage contribution, while bread multigrain is the top contributor to lignans with a 68.3 percentage contribution. The table provides a clear comparison of how different food items contribute to various polyphenol classes.

Navigate back to Table 2.

## Table 3 Long description

The table presents mean differences and 95% confidence intervals for the association between energy-adjusted and energy-unadjusted intake of total polyphenols and their classes with DASS depression subscale scores across four quartiles. It includes data for total polyphenols, flavonoids, lignans, other polyphenols, phenolic acids, and stilbenes. Each polyphenol class is analyzed for energy-adjusted and energy-unadjusted intake, with median values and interquartile ranges provided. The table also includes results from two models: Model 1, unadjusted for any covariate, and Model 2, adjusted for various factors such as age, sex, education, employment, physical activity, smoking status, and alcohol intake. Notable trends and comparisons are highlighted, showing the impact of polyphenol intake on depression scores.

Navigate back to Table 3.

## Table 4 Long description

The table presents data on the mean differences and 95% confidence intervals for the prospective association between energy-adjusted intake of various polyphenol subclasses and DASS depression subscale scores. It includes four quartiles for each polyphenol subclass, with energy-adjusted and energy-unadjusted values in milligrams per day. The table also provides median and interquartile range (IQR) values, as well as results from two models: Model 1, which is unadjusted for any covariate, and Model 2, which is adjusted for age, sex, education, employment, physical activity, smoking status, and alcohol intake. The polyphenol subclasses include anthocyanins, flavanols, flavanones, flavones, flavonols, isoflavonoids, hydroxybenzoic acids, and hydroxycinnamic acids. Notable trends and comparisons are highlighted, such as the significant differences in mean values across quartiles and the adjusted P values for overall differences using the Simes method.

Navigate back to Table 4.
