# Supplementary material for: Polyphenol intake and depressive symptoms in young adults: evidence from a population-based longitudinal study
Source: Br J Nutr. 2025 Dec 26;135(7):711–25. doi: 10.1017/S0007114525105886 (PMC13246595; doi:10.1017/S0007114525105886)
Supplement: Gamage et al. supplementary material [file S0007114525105886sup001.pdf]

Supplementary material

List of tables

STROBE-nut checklist **1**

Table S1. STROBE-nut: An extension of the STROBE statement for nutritional epidemiology Lachat C et al. (2016) STrengthening the Reporting of OBservational studies in Epidemiology – Nutritional Epidemiology (STROBE-nut): an extension of the STROBE statement. *1*

Dietary Questionnaire for Epidemiological Studies version 2 and Dietary Questionnaire for Epidemiological Studies version 3.2 coded for polyphenols **6**

Table S2. Dietary Questionnaire for Epidemiological Studies version 2 coded for total polyphenols, polyphenol classes, and flavonoid and phenolic acid subclasses *6*

Table S3. Dietary Questionnaire for Epidemiological Studies version 3.2 coded for total polyphenols, polyphenol classes, and flavonoid and phenolic acid subclasses *11*

Food contributors to polyphenols **19**

Table S4. The three highest food item contributions to polyphenol subclasses at baseline *19*

Table S5. The three highest food item contributions to total polyphenols, and polyphenol classes at Gen2-22 .... *20*

Table S6. The three highest food item contributions to polyphenol subclasses at Gen2-22 .... *21*

Table S7. The three highest food item contributions to total polyphenols, and polyphenol classes at Gen2-27 .... *22*

Table S8. The three highest food item contributions to polyphenol subclasses at Gen2-27 .... *23*

Association between total polyphenols, and polyphenol classes and depressive symptoms: Sensitivity analyses **24**

Table S9. Mean differences and 95% confidence intervals for the prospective association between energy-adjusted intake of total polyphenols and polyphenol classes and DASS depression subscale scores after excluding participants with a DASS depression subscale cut-off score of ≥ 14 at baseline *24*

Table S10. Mean differences and 95% confidence intervals for the prospective association between energy-adjusted intake of total polyphenols and polyphenol classes and DASS depression subscale scores after excluding participants with a self-diagnosis of depression at baseline *25*

Table S11. Mean differences and 95% confidence intervals for the prospective association between energy-adjusted intake of total polyphenols and polyphenol classes and DASS depression subscale scores after excluding participants with a self-diagnosis of anxiety at baseline *26*

Table S12. Mean differences and 95% confidence intervals for the prospective association between energy-adjusted intake of total polyphenols and polyphenol classes and DASS depression subscale scores after excluding participants with a body mass index of ≥30 kg/m^2 at baseline *27*

Table S13. Mean differences and 95% confidence intervals for the prospective association between energy-unadjusted intake of total polyphenols and polyphenol classes and DASS depression subscale scores *28*

Association between total polyphenols, and polyphenol classes and depressive symptoms: Exploratory analyses **29**

Table S14. Odds ratios (OR) and 95% confidence intervals (CI) for the prospective association between energy-adjusted intake of total polyphenols and polyphenol classes and depression incidence........ 29

Table S15. Mean differences and 95% confidence intervals for the prospective association between energy-adjusted intake of total polyphenols and polyphenol classes and total DASS score............... 30

Table S16. Mean differences and 95% confidence intervals for the prospective association between energy-adjusted intake of total polyphenols and polyphenol classes and DASS depression subscale scores after excluding polyphenols from alcohol 31

Association between total polyphenols, and polyphenol classes and depressive symptoms: Subgroup analyses 33

Table S17. Mean differences and 95% confidence intervals for the prospective association between energy-adjusted intake of total polyphenols and polyphenol classes and DASS depression subscale scores by smoking status at baseline 33

Table S18. Mean differences and 95% confidence intervals for the prospective association between energy-adjusted intake of total polyphenols and polyphenol classes and DASS depression subscale scores by physical activity status at baseline 34

Table S19. Mean differences and 95% confidence intervals for the prospective association between energy-adjusted intake of total polyphenols and polyphenol classes and DASS depression subscale scores by alcohol consumption at baseline 35

Table S20. Mean differences and 95% confidence intervals for the prospective association between energy-adjusted intake of total polyphenols and polyphenol classes and DASS depression subscale scores by sex 36

Association between polyphenol subclasses and depressive symptoms: Sensitivity analyses 37

Table S21. Mean differences and 95% confidence intervals for the prospective association between energy-adjusted intake of polyphenol subclasses and DASS depression subscale scores after excluding participants with a DASS depression subscale cut-off score of ≥ 14 at baseline................ 37

Table S22. Mean differences and 95% confidence intervals for the prospective association between energy-adjusted intake of polyphenol subclasses and DASS depression subscale scores after excluding participants with a self-diagnosis of depression at baseline 38

Table S23. Mean differences and 95% confidence intervals for the prospective association between energy-adjusted intake of polyphenol subclasses and DASS depression subscale scores after excluding participants with a self-diagnosis of anxiety at baseline 39

Table S24. Mean differences and 95% confidence intervals for the prospective association between energy-adjusted intake of polyphenol subclasses and DASS depression subscale scores after excluding participants with a body mass index of ≥30 kg/m² at baseline... 40

Association between polyphenol subclasses and depressive symptoms: Exploratory analyses 43

Table S26. Odds ratios (OR) and 95% confidence intervals (CI) for the prospective association between energy-adjusted intake of polyphenol subclasses and depression incidence 43

Table S27. Mean differences and 95% confidence intervals for the prospective association between energy-adjusted intake of polyphenol subclasses and total DASS score.. 45

Table S28. Mean differences and 95% confidence intervals for the prospective association between energy-adjusted intake of polyphenol subclasses and DASS depression subscale scores after excluding polyphenols from alcohol.. 46

Association between polyphenol subclasses and depressive symptoms: Subgroup analyses 49

Table S29. Mean differences and 95% confidence intervals for the prospective association between energy-adjusted intake of polyphenol subclasses and DASS depression subscale scores by smoking status at baseline.. 49

Table S30. Mean differences and 95% confidence intervals for the prospective association between energy-adjusted intake of polyphenol subclasses and DASS depression subscale scores by physical activity status at baseline 50

Table S31. Mean differences and 95% confidence intervals for the prospective association between energy-adjusted intake of polyphenol subclasses and DASS depression subscale scores by alcohol consumption at baseline 51

Table S32. Mean differences and 95% confidence intervals for the prospective association between energy-adjusted intake of polyphenol subclasses and DASS depression subscale scores by sex 52

STROBE-nut checklist

Table S1. STROBE-nut: An extension of the STROBE statement for nutritional epidemiology Lachat C et al. (2016) STrengthening the Reporting of OBservational studies in Epidemiology – Nutritional Epidemiology (STROBE-nut): an extension of the STROBE statement.

Item	Item nr	STROBE recommendations	Extension for Nutritional Epidemiology studies (STROBE-nut)	Reported on page #
**Title and abstract**	1	(a) Indicate the study's design with a commonly used term in the title or the abstract.<br>(b) Provide in the abstract an informative and balanced summary of what was done and what was found.	**nut-1** State the dietary/nutritional assessment method(s) used in the title, abstract, or keywords.	1
**Introduction**				
Background rationale	2	Explain the scientific background and rationale for the investigation being reported.		1 and 2
Objectives	3	State specific objectives, including any pre-specified hypotheses.		2
**Methods**				
Study design	4	Present key elements of study design early in the paper.		2
Settings	5	Describe the setting, locations, and relevant dates, including periods of recruitment, exposure, follow-up, and data collection.	**nut-5** Describe any characteristics of the study settings that might affect the dietary intake or nutritional status of the participants, if applicable.	2
Participants	6	a) Cohort study—Give the eligibility criteria, and the sources and methods of selection of participants. Describe methods of follow-up.<br>Case-control study—Give the eligibility criteria, and the sources and methods of case ascertainment and control selection. Give the rationale for the choice of cases and controls.<br>Cross-sectional study—Give the eligibility criteria, and the sources and methods of selection of participants.	**nut-6** Report particular dietary, physiological or nutritional characteristics that were considered when selecting the target population.	2

Item	Item nr	STROBE recommendations	Extension for Nutritional Epidemiology studies (STROBE-nut)	Reported on page #
		(b) Cohort study—For matched studies, give matching criteria and number of exposed and unexposed.<br>Case-control study—For matched studies, give matching criteria and the number of controls per case.		
Variables	7	Clearly define all outcomes, exposures, predictors, potential confounders, and effect modifiers. Give diagnostic criteria, if applicable.	**nut-7.1** Clearly define foods, food groups, nutrients, or other food components.<br>**nut-7.2** When using dietary patterns or indices, describe the methods to obtain them and their nutritional properties.	2 to 4
Data sources - measurements	8	For each variable of interest, give sources of data and details of methods of assessment (measurement). Describe comparability of assessment methods if there is more than one group.	**nut-8.1** Describe the dietary assessment method(s), e.g., portion size estimation, number of days and items recorded, how it was developed and administered, and how quality was assured. Report if and how supplement intake was assessed.<br>**nut-8.2** Describe and justify food composition data used. Explain the procedure to match food composition with consumption data. Describe the use of conversion factors, if applicable.<br>**nut-8.3** Describe the nutrient requirements, recommendations, or dietary guidelines and the evaluation approach used to compare intake with the dietary reference values, if applicable.<br>**nut-8.4** When using nutritional biomarkers, additionally use the STROBE Extension for Molecular Epidemiology (STROBE-ME). Report the type of biomarkers used and their usefulness as dietary exposure markers.<br>**nut-8.5** Describe the assessment of nondietary data (e.g., nutritional status and influencing factors) and timing of the	2 to 4

Item	Item nr	STROBE recommendations	Extension for Nutritional Epidemiology studies (STROBE-nut)	Reported on page #
			assessment of these variables in relation to dietary assessment.<br>**nut-8.6** Report on the validity of the dietary or nutritional assessment methods and any internal or external validation used in the study, if applicable.	
Bias	9	Describe any efforts to address potential sources of bias.	**nut-9** Report how bias in dietary or nutritional assessment was addressed, e.g., misreporting, changes in habits as a result of being measured, or data imputation from other sources	2o to 4
Study Size	10	Explain how the study size was arrived at.		2
Quantitative variables	11	Explain how quantitative variables were handled in the analyses. If applicable, describe which groupings were chosen and why.	**nut-11** Explain categorization of dietary/nutritional data (e.g., use of N-tiles and handling of nonconsumers) and the choice of reference category, if applicable.	2 to 4
Statistical Methods	12	(a) Describe all statistical methods, including those used to control for confounding<br>(b) Describe any methods used to examine subgroups and interactions.<br>(c) Explain how missing data were addressed.<br>(d) Cohort study—If applicable, explain how loss to follow-up was addressed.<br>Case-control study—If applicable, explain how matching of cases and controls was addressed.<br>Cross-sectional study—If applicable, describe analytical methods taking account of sampling strategy.<br>(e) Describe any sensitivity analyses.	**nut-12.1** Describe any statistical method used to combine dietary or nutritional data, if applicable.<br>**nut-12.2** Describe and justify the method for energy adjustments, intake modeling, and use of weighting factors, if applicable.<br>**nut-12.3** Report any adjustments for measurement error, i.e,. from a validity or calibration study.	2 to 4
**Results**				
Participants	13	(a) Report the numbers of individuals at each stage of the study—e.g., numbers potentially eligible,	**nut-13** Report the number of individuals excluded based on missing, incomplete or implausible dietary/nutritional data.	5 and 6

Item	Item nr	STROBE recommendations	Extension for Nutritional Epidemiology studies (STROBE-nut)	Reported on page #
		examined for eligibility, confirmed eligible, included in the study, completing follow-up, and analyzed.<br>(b) Give reasons for non-participation at each stage.<br>(c) Consider use of a flow diagram.		
Descriptive data	14	(a) Give characteristics of study participants (e.g., demographic, clinical, social) and information on exposures and potential confounders<br>(b) Indicate the number of participants with missing data for each variable of interest<br>(c) Cohort study—Summarize follow-up time (e.g., average and total amount)	**nut-14** Give the distribution of participant characteristics across the exposure variables if applicable. Specify if food consumption of total population or consumers only were used to obtain results.	2 and 4
Outcome data	15	Cohort study—Report numbers of outcome events or summary measures over time.<br>Case-control study—Report numbers in each exposure category, or summary measures of exposure.<br>Cross-sectional study—Report numbers of outcome events or summary measures.		Table 1
Main results	16	(a) Give unadjusted estimates and, if applicable, confounder-adjusted estimates and their precision (e.g., 95% confidence interval).<br>Make clear which confounders were adjusted for and why they were included.<br>(b) Report category boundaries when continuous variables were categorized.<br>(c) If relevant, consider translating estimates of relative risk into absolute risk for a meaningful time period.	**nut-16** Specify if nutrient intakes are reported with or without inclusion of dietary supplement intake, if applicable.	6<br>Table 3 and Table 4

Item	Item nr	STROBE recommendations	Extension for Nutritional Epidemiology studies (STROBE-nut)	Reported on page #
Other analyses	17	Report other analyses done—e.g., analyses of subgroups and interactions and sensitivity analyses.	**nut-17** Report any sensitivity analysis (e.g., exclusion of misreporters or outliers) and data imputation, if applicable.	Supplementary Tables S9 to S30
**Discussion**				
Key results	18	Summarize key results with reference to study objectives.		11
Limitation	19	Discuss limitations of the study, taking into account sources of potential bias or imprecision. Discuss both direction and magnitude of any potential bias.	**nut-19** Describe the main limitations of the data sources and assessment methods used and implications for the interpretation of the findings.	12 and 13
Interpretation	20	Give a cautious overall interpretation of results considering objectives, limitations, multiplicity of analyses, results from similar studies, and other relevant evidence.	**nut-20** Report the nutritional relevance of the findings, given the complexity of diet or nutrition as an exposure.	11 to 13
Generalizability	21	Discuss the generalizability (external validity) of the study results.		12
**Other information**				
Funding	22	Give the source of funding and the role of the funders for the present study and, if applicable, for the original study on which the present article is based.		13
Ethics			**nut-22.1** Describe the procedure for consent and study approval from ethics committee(s).	2
Supplementary material			**nut-22.2** Provide data collection tools and data as online material or explain how they can be accessed.	2

Dietary Questionnaire for Epidemiological Studies version 2 and Dietary Questionnaire for Epidemiological Studies version 3.2 coded for polyphenols

Table S2. Dietary Questionnaire for Epidemiological Studies version 2 coded for total polyphenols, polyphenol classes, and flavonoid and phenolic acid subclasses

Food item	Total polyphenols	Flavonoids	Lignans	Other polyphenols	Phenolic acids	Stilbenes	Anthocyanins	Flavanols	Flavanones	Flavones	Flavonols	Isoflavonoids	Hydroxybenzoic acids	Hydroxycinnamic acids
	mg per 100g													
Milk – full cream														
Milk – reduced fat														
Milk – skim														
Milk – soy	18.0543	18.0223	0.0314	0.0006								18.0223		
Bread – high-fibre white bread	0.0763		0.0763											
Bread – white	0.0763		0.0763											
Bread – wholemeal	24.8022		0.0855	24.7167										
Bread – rye	67.3601		0.1828	61.9373	5.2400								0.5700	4.6700
Bread – multigrain	42.7864	12.8494	12.4042	12.4141	5.1187					1.8244		11.0249	0.0066	5.1100
Margarine of any kind	5.6397	0.0505	0.0144	5.5568	0.0181					0.0505			0.0102	0.0057
Polyunsaturated margarine	0.3297	0.0012	0.0008	0.3264	0.0012					0.0012			0.0004	0.0009
Monounsaturated margarine	8.6217	0.0782	0.0220	8.4940	0.0275					0.0782			0.0158	0.0083
Margarine and butter blends														
Butter														
Hard cheeses (e.g., parmesan, romano)														
Firm cheeses (e.g., cheddar, edam)														
Soft cheeses (e.g., camembert, brie)														
Ricotta or cottage cheese														

Food item	Total polyphenols	Flavonoids	Lignans	Other polyphenols	Phenolic acids	Stilbenes	Anthocyanins	Flavanols	Flavanones	Flavones	Flavonols	Isoflavonoids	Hydroxybenzoic acids	Hydroxycinnamic acids
	mg per 100g													
Cream cheese														
Low fat cheese														
Sugar														
Eggs														
All Bran	820.8479		2.3657	397.6299	420.8523								5.9273	414.9249
Sultana Bran, Fibre Plus, Bran Flakes	422.8550	93.9121	1.0478	158.1697	169.5804	0.1450		53.8712		38.3841	1.4861		1.8828	167.6976
Weet Bix, Vita Brits, Weeties	203.2206	75.9953	0.5374	63.0859	63.6020					75.9953				63.6020
Cornflakes, Nutrigrain, Special K	78.5071	7.3419	0.1317	6.1259	64.9076					7.3419			0.2076	64.6680
Porridge	4.8885		0.1089	0.0425	4.7372								0.0935	4.6437
Muesli	155.5029	48.7801	2.3178	38.6036	65.7429	0.0586	1.8327	41.5694	0.0191	4.0275	1.1246	0.0005	2.1530	63.5898
Rice	3.3630				3.3630								0.0976	3.2653
Pasta or noodles	2.5266		0.0018	2.5248										
Crackers, crispbreads or dry biscuits	20.6955	0.2658	0.8104	10.9184	8.7009				0.0503	0.0465	0.1549	0.0033	0.1734	8.5268
Sweet biscuits	55.8116	54.1909	0.0140	0.2803	1.3227	0.0037	0.3236	53.5370	0.0280	0.2497	0.0490		0.5620	0.7607
Cakes, sweet pies, tarts or other sweet pastries	74.0337	65.9784	0.0685	2.0260	5.9333	0.0275	2.5224	57.9090	0.3974	4.0952	1.0029	0.0331	1.7032	4.2300
Meat pies, pasties, quiche or other savoury pastries	12.7886	9.9278	0.0577	0.6108	2.1913	0.0009	0.0411	0.0537	0.2984	3.5284	5.9953	0.0095	0.2768	1.9144
Pizza	6.4084	2.6298	0.0516	1.9612	1.7659	0.0000	0.3613	0.0076	0.0449	0.1623	2.0522	0.0001	0.2703	1.3585
Hamburger with a bun	4.8920	3.8108	0.2174	0.4248	0.4388	0.0003	0.0519	0.0328	0.0117	0.0939	3.5566	0.0635	0.0561	0.3826
Chocolate	407.8011	403.9177	0.0008	0.0006	3.8616	0.0205	0.4821	399.7656	0.0122	0.0004	3.6574		0.0615	3.8001
Flavoured milk	6.7464	6.4831	0.0014	0.0013	0.2597	0.0009	0.1716	6.2693			0.0422		0.0489	0.2109
Nuts	117.6828	90.9215	0.2240	0.0095	26.5125	0.0152		84.0257	0.0678	0.0001	6.8280		24.8132	1.6993

Food item	Total polyphenols	Flavonoids	Lignans	Other polyphenols	Phenolic acids	Stilbenes	Anthocyanins	Flavanols	Flavanones	Flavones	Flavonols	Isoflavonoids	Hydroxybenzoic acids	Hydroxycinnamic acids
	mg per 100g													
Peanut butter	11.2157	11.1000	0.0665			0.0492		11.1000						
Corn chips, potato crisps, Twisties, etc.	20.3242	0.0970	0.0027	0.0612	20.1632				0.0000	0.0167	0.0803		0.0544	20.0998
Jam, honey, marmalade or syrups	10.3379	6.5211	0.0172		3.7971	0.0025	1.0855	2.4806	2.6590		0.2959		2.8752	0.9219
Vegemite, marmite or promite														
Ice cream	23.9094	23.2591	0.0022	0.0134	0.6339	0.0008	0.1567	22.9907		0.0994	0.0123		0.3308	0.3031
Yoghurt	7.8136	6.6252	0.0101	0.0000	1.1732	0.0051	2.7158	3.6269			0.2824		0.4729	0.7003
Beef														
Veal														
Chicken														
Lamb														
Pork														
Bacon														
Ham														
Corned beef, luncheon meats or salami														
Sausages or frankfurters														
Fish steamed, grilled or baked														
Fish fried (include takeaway)	2.6141	1.2080	0.0265	1.0234	0.3563					1.2080			0.0088	0.3469
Fish tinned (e.g., salmon, tuna, sardines)	2.3078	1.7441	0.0031	0.4310	0.1295	0.0000		0.0024	0.0032	0.0069	1.7316		0.0323	0.0972
Fruit tinned or frozen (any kind)	128.1038	98.2019	0.1354		29.7287	0.0378	47.8694	44.4616			5.8709		16.9538	12.7748
Fruit juice	35.9677	28.6867	0.0115	0.4282	6.8379	0.0034	1.1820	2.2563	21.7608	1.8724	0.5544		0.1946	6.6434

Food item	Total polyphenols	Flavonoids	Lignans	Other polyphenols	Phenolic acids	Stilbenes	Anthocyanins	Flavanols	Flavanones	Flavones	Flavonols	Isoflavonoids	Hydroxybenzoic acids	Hydroxycinnamic acids
	mg per 100g													
Oranges or other citrus fruit	49.6834	48.2784	0.0647		1.3403				47.6890	0.3400	0.2494		0.0038	1.3364
Apples	143.3612	124.3481	0.0028		19.0102		0.9299	110.9957		0.1707	6.8644		1.1221	17.8882
Pears	16.8716	4.8223	0.1941	0.0467	11.8086			4.0489			0.7733		0.5400	11.2686
Bananas	6.2666	5.2509	0.0124		1.0033			5.2509					1.0033	
Watermelon, cantaloupe/rockmelon, honeydew, etc.	1.2951	1.2311	0.0640							1.2311				
Pineapple	0.1222		0.1222											
Strawberries	239.8108	226.3118	0.4096		12.7394	0.3500	73.0020	147.5632			5.7466		5.6691	7.0703
Apricots	30.7889	20.2901	0.4638		10.0350			19.3329			0.9573			10.0350
Peaches or nectarines	22.4151	7.7199	0.1398		14.5554		0.2847	7.0634			0.3719			14.5554
Mango or paw paw	6.0665	6.0600	0.0065					6.0600						
Avocado	8.0381	7.0271	1.0110					7.0271						
Potatoes, roasted or fried (include hot chips)	24.3781	3.4191	0.0180	1.5923	19.3488					0.0200	3.3991		3.2143	16.1335
Potatoes cooked without fat	28.4377	0.0580	0.0301		28.3497					0.0020	0.0560			28.3497
Tomato sauce, tomato paste or dried tomatoes	21.3513	1.4572	0.2968		19.5973				0.6841		0.7731			19.5973
Fresh or tinned tomatoes	4.2007	0.2876	0.0460		3.8672				0.1350		0.1526			3.8672
Peppers (capsicum)	2.3308	1.6232	0.1244		0.5832					0.7907	0.8325			0.5832
Lettuce, endive, other salad greens	18.6687	15.3814	0.0244	0.0487	3.2142		0.0310			1.5961	13.7543		0.0221	3.1920
Cucumber	0.1572	0.0972	0.0600							0.0020	0.0952			
Celery	2.5920		0.0060	2.5860										
Beetroot	0.5071	0.5000	0.0071							0.3660	0.1340			
Carrots	14.9574	0.7483	0.1563		14.0528					0.2733	0.4750		0.0500	14.0029

Food item	Total polyphenols	Flavonoids	Lignans	Other polyphenols	Phenolic acids	Stilbenes	Anthocyanins	Flavanols	Flavanones	Flavones	Flavonols	Isoflavonoids	Hydroxybenzoic acids	Hydroxycinnamic acids
	mg per 100g													
Cabbage or brussels sprouts	1.6863	1.0889	0.5974							0.1578	0.9311			
Cauliflower	8.6800	1.2833	0.2117		7.1850					0.2000	1.0833		2.2650	4.9200
Broccoli	46.6699	27.8000	1.3452		17.5246						27.8000			17.5246
Spinach or silverbeet	135.5729	33.8532	0.0267		101.6930					0.2213	33.6319		27.4737	74.2193
Peas	0.1533	0.0200	0.1333					0.0200						
Green beans	6.9318	6.6351	0.2966					1.0923			5.5428			
Bean sprouts or alfalfa sprouts														
Baked beans	48.7637	24.8528	0.0688	0.0025	23.8395	0.0000		0.0504	0.0052	0.0001	24.5513	0.2457	0.0031	23.8365
Soybeans, soybean curd or tofu	101.0417	100.9052	0.1302	0.0063								100.9052		
Other beans (include chickpeas, lentils, etc.)	171.3637	144.7029	0.0711		26.5717	0.0180	5.1358	120.8065		0.1900	18.1506	0.4200	0.0920	26.4797
Pumpkin	1.7391	1.6300	0.1091							1.6300				
Onion or leeks	32.0260	31.5273	0.0467		0.4519		0.5143				31.0131		0.4519	
Garlic	0.4931		0.4931											
Mushrooms														
Zucchini	1.4488	1.3240	0.1248								1.3240			
Light beer	0.8876	0.1199		0.2773	0.4905			0.1086	0.0110				0.3117	0.1788
Heavy beer	1.8652	0.6388		0.0105	1.2158			0.3871	0.2347			0.0020	0.6833	0.5325
Red wine	101.4472	76.3841	0.1282	4.3668	17.1529	3.4152	22.3383	40.8913	0.8600		6.8549		7.0041	9.9887
White wine	10.4107	3.3672	0.0505	0.8300	5.4013	0.7617	0.0388	2.0554	0.2300		0.4740		2.4780	2.8283
Fortified wines, port, sherry, etc.	16.0115	3.6300	0.0250	6.0906	6.2659			3.6200			0.0100		1.4117	4.8542
Spirits	0.7427	0.1643	0.0013	0.0788	0.4984		0.1426	0.0001	0.0203	0.0007	0.0007		0.3255	0.1728

Table S3. Dietary Questionnaire for Epidemiological Studies version 3.2 coded for total polyphenols, polyphenol classes, and flavonoid and phenolic acid subclasses

Food item	Total polyphenols	Flavonoids	Lignans	Other polyphenols	Phenolic acids	Stilbenes	Anthocyanins	Flavanols	Flavanones	Flavones	Flavonols	Isoflavonoids	Hydroxybenzoic acids	Hydroxycinnamic acids
	mg per 100g													
Milk – full cream														
Milk – reduced fat														
Milk – skim														
Milk – soy	18.0543	18.0223	0.0314	0.0006								18.0223		
Flavoured milk	6.7464	6.4831	0.0014	0.0013	0.2597	0.0009	0.1716	6.2693			0.0422		0.0489	0.2109
Milk – rice/oat/other	1.9606		0.0340	0.0129	1.9137								0.0320	1.8817
Diet soft drinks														
Soft drinks	0.2612	0.2552	0.0000	0.0009	0.0051				0.2509	0.0038	0.0005			0.0051
Water														
Bread – high-fibre white bread	0.0763		0.0763											
Bread – white	0.0763		0.0763											
Bread – wholemeal	24.8022		0.0855	24.7167										
Bread – rye	67.3601		0.1828	61.9373	5.2400								0.5700	4.6700
Bread – multigrain	42.7864	12.8494	12.4042	12.4141	5.1187					1.8244		11.0249	0.0066	5.1100
Margarine and butter blends														
Butter														
Eggs														
Sugar														
Sweet biscuits	55.8116	54.1909	0.0140	0.2803	1.3227	0.0037	0.3236	53.5370	0.0280	0.2497	0.0490		0.5620	0.7607
Cakes, sweet pies, tarts, other sweet pastries	74.0337	65.9784	0.0685	2.0260	5.9333	0.0275	2.5224	57.9090	0.3974	4.0952	1.0029	0.0331	1.7032	4.2300
Rice	3.3630				3.3630								0.0976	3.2653
Pasta or noodles	2.5266		0.0018	2.5248										

Food item	Total polyphenols	Flavonoids	Lignans	Other polyphenols	Phenolic acids	Stilbenes	Anthocyanins	Flavanols	Flavanones	Flavones	Flavonols	Isoflavonoids	Hydroxybenzoic acids	Hydroxycinnamic acids
	mg per 100g													
Pizza	6.4084	2.6298	0.0516	1.9612	1.7659	0.0000	0.3613	0.0076	0.0449	0.1623	2.0522	0.0001	0.2703	1.3585
Jam, honey, marmalade, or syrups	10.3379	6.5211	0.0172		3.7971	0.0025	1.0855	2.4806	2.6590		0.2959		2.8752	0.9219
Vegemite, marmite, or promite														
Ice cream	23.9094	23.2591	0.0022	0.0134	0.6339	0.0008	0.1567	22.9907		0.0994	0.0123		0.3308	0.3031
Yoghurt	7.8136	6.6252	0.0101	0.0000	1.1732	0.0051	2.7158	3.6269			0.2824		0.4729	0.7003
Chicken														
Lamb														
Pork														
Bacon														
Sausages or frankfurters														
Fish steamed, grilled, or baked														
Fish fried (include take away)	2.6141	1.2080	0.0265	1.0234	0.3563					1.2080			0.0088	0.3469
Fish tinned (e.g., salmon, tuna, sardines)	2.3078	1.7441	0.0031	0.4310	0.1295	0.0000		0.0024	0.0032	0.0069	1.7316		0.0323	0.0972
Fruit tinned or frozen (any kind)	128.1038	98.2019	0.1354		29.7287	0.0378	47.8694	44.4616			5.8709		16.9538	12.7748
Oranges or other citrus fruit	49.6834	48.2784	0.0647		1.3403				47.6890	0.3400	0.2494		0.0038	1.3364
Apples	143.3612	124.3481	0.0028		19.0102		0.9299	110.9957		0.1707	6.8644		1.1221	17.8882
Pears	16.8716	4.8223	0.1941	0.0467	11.8086			4.0489			0.7733		0.5400	11.2686
Bananas	6.2666	5.2509	0.0124		1.0033			5.2509					1.0033	
Pineapple	0.1222		0.1222											
Strawberries	239.8108	226.3118	0.4096		12.7394	0.3500	73.0020	147.5632			5.7466		5.6691	7.0703
Apricots	30.7889	20.2901	0.4638		10.0350			19.3329			0.9573			10.0350
Peaches or nectarines	22.4151	7.7199	0.1398		14.5554		0.2847	7.0634			0.3719			14.5554

Food item	Total polyphenols	Flavonoids	Lignans	Other polyphenols	Phenolic acids	Stilbenes	Anthocyanins	Flavanols	Flavanones	Flavones	Flavonols	Isoflavonoids	Hydroxybenzoic acids	Hydroxycinnamic acids
	mg per 100g													
Mango or paw paw	6.0665	6.0600	0.0065					6.0600						
Avocado	8.0381	7.0271	1.0110					7.0271						
Potatoes, roasted or fried (include hot chips)	24.3781	3.4191	0.0180	1.5923	19.3488					0.0200	3.3991		3.2143	16.1335
Potatoes cooked without fat	28.4377	0.0580	0.0301		28.3497					0.0020	0.0560			28.3497
Peppers (capsicum)	2.3308	1.6232	0.1244		0.5832					0.7907	0.8325			0.5832
Cucumber	0.1572	0.0972	0.0600							0.0020	0.0952			
Celery	2.5920		0.0060	2.5860										
Beetroot	0.5071	0.5000	0.0071							0.3660	0.1340			
Carrots	14.9574	0.7483	0.1563		14.0528					0.2733	0.4750		0.0500	14.0029
Cabbage or brussels sprouts	1.6863	1.0889	0.5974							0.1578	0.9311			
Cauliflower	8.6800	1.2833	0.2117		7.1850					0.2000	1.0833		2.2650	4.9200
Broccoli	46.6699	27.8000	1.3452		17.5246						27.8000			17.5246
Spinach or silverbeet	135.5729	33.8532	0.0267		101.6930					0.2213	33.6319		27.4737	74.2193
Peas	0.1533	0.0200	0.1333					0.0200						
Green beans	6.9318	6.6351	0.2966					1.0923			5.5428			
Baked beans	48.7637	24.8528	0.0688	0.0025	23.8395	0.0000		0.0504	0.0052	0.0001	24.5513	0.2457	0.0031	23.8365
Pumpkin	1.7391	1.6300	0.1091							1.6300				
Onion or leeks	32.0260	31.5273	0.0467		0.4519		0.5143				31.0131		0.4519	
Mushrooms														
Zucchini	1.4488	1.3240	0.1248								1.3240			
Olive oil	50.1000	0.6339	0.1071	49.0782	0.2808					0.6339			0.1612	0.0852
Canola oil														
Vegetable oil	5.0577	0.0634	0.0107	4.9545	0.0291					0.0634			0.0164	0.0093
Olive oil margarine	18.6131	0.0807	0.0578	18.4747						0.0807				

Food item	Total polyphenols	Flavonoids	Lignans	Other polyphenols	Phenolic acids	Stilbenes	Anthocyanins	Flavanols	Flavanones	Flavones	Flavonols	Isoflavonoids	Hydroxybenzoic acids	Hydroxycinnamic acids
	mg per 100g													
Margarine – canola (e.g., Gold'n Canola, Meadow Lea Canola)														
Margarine – sterol margarine or margarine to lower cholesterol (e.g., Pro-activ, Meadow Lea Heart Plus, Logicol)	0.0620			0.0607	0.0014								0.0004	0.0010
Margarine – olive oil (e.g., Bertolli, Olive Grove, Olivani)	18.6131	0.0807	0.0578	18.4747						0.0807				
Margarine – polyunsaturated	0.3297	0.0012	0.0008	0.3264	0.0012					0.0012			0.0004	0.0009
Bread – soy and linseed	90.6638	11.5644	63.3198	11.1727	4.6069					1.6420		9.9224	0.0059	4.5990
Bread – gluten free	32.1938	2.0233	0.0296	0.5414	29.5996						2.0233		1.6149	27.9698
Cereal – wheat biscuits or wheat flakes (e.g., Weet Bix, Vita Brits, Weeties)	203.2206	75.9953	0.5374	63.0859	63.6020					75.9953				63.6020
Cereal – cornflakes	136.9350				136.9350								0.2520	136.6020
Cereal – bran based cereals (e.g., All-Bran, Sultana Bran)	556.1801	40.3101	1.5544	249.5002	264.7587	0.0566		18.7007	0.0021	20.2531	0.5758	0.7784	3.4886	261.2701
Cereal – untoasted muesli	191.5006	57.2168	1.7295	51.2264	81.2551	0.0728	2.8540	46.3228	0.0219	6.4403	1.3545	0.0007	2.6832	78.5718
Cereal – toasted muesli	134.6137	45.7412	1.7138	30.2700	56.8379	0.0509	1.0151	41.4307	0.0184	2.0621	1.0015	0.0004	1.8306	55.0072
Cereal – mixed grain (e.g., Just Right, Sustain, Light Tasty, Uncles Toby's Plus varieties)	229.1644	68.2622	1.3406	66.7173	92.7701	0.0742	2.5304	33.0874	0.0027	31.5361	0.9309		1.2191	91.5428
Cereal – sugary (e.g., Crunchy Nut	95.3764	34.1255	0.0030	0.0045	61.2415	0.0020		34.1122				0.0133	0.6466	60.5601

Food item	Total polyphenols	Flavonoids	Lignans	Other polyphenols	Phenolic acids	Stilbenes	Anthocyanins	Flavanols	Flavanones	Flavones	Flavonols	Isoflavonoids	Hydroxybenzoic acids	Hydroxycinnamic acids
	mg per 100g													
Cornflakes, Coco Pops, Frosties)														
Cereal – rice-based (e.g., Rice Bubbles, puffed rice, rice flakes)	6.6612				6.6612								0.2548	6.4064
Cereal – Special K	31.1774	8.8876	0.1594	7.4155	14.7148					8.8876			0.2005	14.5143
Porridge – average	4.8885		0.1089	0.0425	4.7372								0.0935	4.6437
Cereal – Nutrigrain	67.4090	13.1382	0.2357	10.9621	43.0731					13.1382			0.1702	42.8876
Wholemeal crackers or dry biscuits	23.9322	0.0021	1.3241	16.6792	5.9269							0.0021	0.0925	5.8344
Other biscuits	0.0125		0.0125											
Margarine on cooked vegetables	5.6397	0.0505	0.0144	5.5568	0.0181					0.0505			0.0102	0.0057
Butter on cooked vegetables														
Oil on cooked vegetables	5.0577	0.0634	0.0107	4.9545	0.0291					0.0634			0.0164	0.0093
Low calorie/fat dressing	3.6581	1.2602	0.0083	0.7499	1.6388	0.0009		0.3559	0.3266	0.0716	0.5060		0.8490	0.7898
Mayonnaise	1.3439	0.3118	0.0000	0.3406	0.6911	0.0004		0.1684	0.1157	0.0169	0.0108		0.3948	0.2963
Oil and vinegar dressing	12.0214	3.1247	0.0188	7.4845	1.3926	0.0008		0.3173	2.3234	0.1746	0.2285	0.0808	0.7701	0.6180
Other cheese														
Ricotta or cottage cheese														
Cream or sour cream														
Beef and veal														
Processed meat	1.5575	1.1816	0.0019	0.0655	0.2573	0.0512	0.3351	0.6134	0.0129	0.0141	0.1246		0.1051	0.1498
Other seafood														
Berries not including strawberries	243.9057	180.6099	0.1564		63.0822	0.0572	82.8945	81.6613			16.0541		34.4808	28.6014
Cherries	269.7575	181.9532			87.8043		171.4175	9.4157			1.1200			87.8043

Food item	Total polyphenols	Flavonoids	Lignans	Other polyphenols	Phenolic acids	Stilbenes	Anthocyanins	Flavanols	Flavanones	Flavones	Flavonols	Isoflavonoids	Hydroxybenzoic acids	Hydroxycinnamic acids
	mg per 100g													
Figs	0.1200	0.1200						0.1200						
Plums	378.1021	288.8467	0.1142		89.1412		47.7832	234.2218			6.8417		0.0675	89.0737
Grapes	112.4839	109.2445	0.0479		2.9593	0.2321	27.5823	78.8826			2.7796		0.0005	2.9588
Orange juice	46.2973	41.2611	0.0088	0.7405	4.2870				37.6338	3.2382	0.3891			4.2870
Other fruit juice	38.7536	26.3561	0.0361	0.2301	12.1135	0.0178	1.9010	10.8629	10.8964	0.4979	0.9317		3.4204	8.6930
Cantaloupe/rockmelon or honeydew	0.0423		0.0423											
Watermelon	1.9299	1.8400	0.0899							1.8400				
Kiwi fruit	9.2297	9.0890	0.1408					8.3456		0.7433				
Dried apricots	0.6318		0.6318											
Dried fruit	242.4322	230.3813	0.1509		11.3751	0.5249	45.7131	174.5012	4.0037	0.0635	6.0997		0.0817	11.2935
Fresh tomatoes (raw or cooked)	4.2007	0.2876	0.0460		3.8672				0.1350		0.1526			3.8672
Canned tomatoes and tomato juice	4.2909	0.2967	0.0460		3.9481				0.1593		0.1374			3.9481
Tomato products including puree, paste or pasta sauce	13.0110	1.0030	0.2496		11.7584				0.4105		0.5925			11.7584
Iceberg lettuce	8.0834	4.2917	0.0118		3.7800					0.3983	3.8933			3.7800
Other lettuce and salad leaves (e.g., rocket, spinach, endive, chicory)	77.0467	22.4839	0.0223		54.5405		0.3209			0.3650	21.7980		18.0789	36.4615
Asian greens (e.g., bok choy)	54.4000	54.4000								5.7000	48.7000			
Coleslaw	8.1143	6.0207	0.3024	0.4679	1.3232	0.0000		0.0193	0.0057	5.4903	0.5055		0.0496	1.2736
Sweet potato	0.2974		0.2974											
Asparagus	23.6505	23.1950	0.4555								23.1950			
Eggplant	1.5958		0.1058		1.4900								0.8100	0.6800
Garlic	0.4931		0.4931											

Food item	Total polyphenols	Flavonoids	Lignans	Other polyphenols	Phenolic acids	Stilbenes	Anthocyanins	Flavanols	Flavanones	Flavones	Flavonols	Isoflavonoids	Hydroxybenzoic acids	Hydroxycinnamic acids
	mg per 100g													
Sweetcorn	57.3553	0.0027	0.0208		57.3318		0.0027						0.6438	56.6881
Dried beans, dried peas, chick peas or lentils	171.3637	144.7029	0.0711		26.5717	0.0180	5.1358	120.8065		0.1900	18.1506	0.4200	0.0920	26.4797
Olives	496.6056	80.2005	0.0538	238.4389	177.9124		41.4868			13.9993	24.7144		27.9507	132.7500
Pastries with cheese (e.g., quiche, spanakopita)	10.3144	8.6722	0.0451	0.2720	1.3251	0.0000	0.0486	0.2100	0.0014	2.6284	5.7835	0.0002	0.2965	1.0285
Pastries with meat (e.g., meat pies, sausage rolls)	12.6739	9.1716	0.1547	0.8270	2.5199	0.0008	0.0316	0.0097	0.2729	3.7004	5.1247	0.0310	0.2400	2.2796
Tomato sauce or ketchup	0.0080		0.0080											
Chocolate or confectionery containing chocolate	407.8011	403.9177	0.0008	0.0006	3.8616	0.0205	0.4821	399.7656	0.0122	0.0004	3.6574		0.0615	3.8001
Other confectionery	24.6721	22.3479	0.0029	0.1508	2.1695	0.0009	0.0147	21.8637	0.0776	0.2200	0.1694		0.1043	2.0641
Peanut butter	11.2157	11.1000	0.0665			0.0492		11.1000						
Other nuts	174.9353	126.0601	0.3158	0.0140	48.5454			112.8164	0.0997	0.0002	13.1437		48.5453	0.0000
Corn chips, potato crisps, twisties	20.3242	0.0970	0.0027	0.0612	20.1632				0.0000	0.0167	0.0803		0.0544	20.0998
Tea	88.0995	71.3301	0.0211		16.7482			63.1591			8.1710		14.1211	2.6272
Herbal tea	13.7664	5.2321	0.0003	0.4986	8.0354			2.4600		2.1200	0.6521			8.0354
Coffee	214.7585		0.0102	2.5883	212.1600									212.1600
Coffee substitute														
Light beer	0.8876	0.1199		0.2773	0.4905			0.1086	0.0110				0.3117	0.1788
Heavy beer	1.8652	0.6388		0.0105	1.2158			0.3871	0.2347			0.0020	0.6833	0.5325
Red wine	101.4472	76.3841	0.1282	4.3668	17.1529	3.4152	22.3383	40.8913	0.8600		6.8549		7.0041	9.9887
White wine	10.4107	3.3672	0.0505	0.8300	5.4013	0.7617	0.0388	2.0554	0.2300		0.4740		2.4780	2.8283
Premixed spirits	0.4778	0.4063	0.0003	0.0132	0.0580				0.4063				0.0580	

Food item	Total polyphenols	Flavonoids	Lignans	Other polyphenols	Phenolic acids	Stilbenes	Anthocyanins	Flavanols	Flavanones	Flavones	Flavonols	Isoflavonoids	Hydroxybenzoic acids	Hydroxycinnamic acids
	mg per 100g													
Spirits	0.7427	0.1643	0.0013	0.0788	0.4984		0.1426	0.0001	0.0203	0.0007	0.0007		0.3255	0.1728

Food contributors to polyphenols

Table S4. The three highest food item contributions to polyphenol subclasses at baseline

Polyphenol subclass	Top three contributors	Percentage contribution
**Anthocyanins**	Strawberries	36.9%
	Tinned or frozen fruit (any kind)	27.8%
	Red wine	18.5%
**Flavanols**	Black tea	30.5%
	Apples	20.1%
	Chocolate	15.1%
**Flavanones**	Fruit juice	57.5%
	Oranges or other citrus fruit	40.3%
	Heavy beer	0.9%
**Flavones**	Weet Bix, Vita Brits, Weeties	46.4%
	Fruit juice	13.9%
	Sultana Bran, Fibre Plus, Bran Flakes	10.2%
**Flavonols**	Black tea	33.2%
	Apples	9.0%
	Broccoli	6.9%
**Isoflavonoids**	Soybeans, soybean curd or tofu	36.4%
	Milk – soy	32.4%
	Bread – multigrain	30.4%
**Hydroxybenzoic acids**	Black tea	57.9%
	Green tea	10.6%
	Tinned or frozen fruit (any kind)	5.7%
**Hydroxycinnamic acids**	Coffee	79.5%
	Apples	2.1%
	Fruit juice	1.6%

Table S5. The three highest food item contributions to total polyphenols, and polyphenol classes at Gen2-22

Polyphenol class	Top three contributors	Percentage contribution
**Total polyphenols**	Coffee	39.6%
	Black tea	12.8%
	Apples	7.4%
**Flavonoids**	Black tea	23.4%
	Apples	14.8%
	Chocolate	11.6%
**Lignans**	Bread – multigrain	66.1%
	Muesli	4.8%
	Broccoli	3.8%
**Other polyphenols**	Weet Bix, Vita Brits, Weeties	14.4%
	Coffee	12.5%
	Sultana Bran, Fibre Plus, Bran Flakes	12.1%
**Phenolic Acids**	Coffee	74.4%
	Black tea	5.1%
	Apples	1.9%
**Stilbenes**	Red wine	73.7%
	White wine	21.3%
	Strawberries	3.1%

Table S6. The three highest food item contributions to polyphenol subclasses at Gen2-22

Polyphenol subclass	Top three contributors	Percentage contribution
**Anthocyanins**	Strawberries	33.4%
	Tinned or frozen fruit (any kind)	27.3%
	Red wine	25.1%
**Flavanols**	Black tea	29.1%
	Apples	18.9%
	Chocolate	16.4%
**Flavanones**	Fruit juice	53.7%
	Oranges or other citrus fruit	43.2%
	Heavy beer	1.2%
**Flavones**	Weet Bix, Vita Brits, Weeties	47.7%
	Fruit juice	11.1%
	Sultana Bran, Fibre Plus, Bran Flakes	8.1%
**Flavonols**	Black tea	30.9%
	Apples	8.3%
	Spinach or silverbeet	6.9%
**Isoflavonoids**	Soybeans, soybean curd or tofu	36.6%
	Milk – soy	35.7%
	Bread – multigrain	26.9%
**Hydroxybenzoic acids**	Black tea	54.5%
	Green tea	10.6%
	Tinned or frozen fruit (any kind)	5.9%
**Hydroxycinnamic acids**	Coffee	80.9%
	Apples	1.9%
	Potatoes cooked without fat	1.4%

Table S7. The three highest food item contributions to total polyphenols, and polyphenol classes at Gen2-27

Polyphenol class	Top three contributors	Percentage contribution
**Total polyphenols**	Coffee	50.3%
	Tea	9.3%
	Dried beans, dried peas, chickpeas or lentils	6.5%
**Flavonoids**	Tea	21.4%
	Dried beans, dried peas, chickpeas or lentils	15.5%
	Apples	12.4%
**Lignans**	Bread – multigrain	42.7%
	Bread – soy and linseed	24.1%
	Broccoli	5.7%
**Other polyphenols**	Coffee	19.1%
	Olives	15.7%
	Olive oil	14.0%
**Phenolic Acids**	Coffee	81.2%
	Tea	2.9%
	Spinach or silverbeet	1.9%
**Stilbenes**	Red wine	81.3%
	White wine	12.1%
	Strawberries	3.0%

Table S8. The three highest food item contributions to polyphenol subclasses at Gen2-27

**Polyphenol subclass**	**Top three contributors**	**Percentage contribution**
**Anthocyanins**	Strawberries	25.3%
	Red wine	21.4%
	Berries not including strawberries	15.8%
**Flavanols**	Tea	27.1%
	Dried beans, dried peas, chickpeas or lentils	18.5%
	Apples	15.7%
**Flavanones**	Orange juice	51.6%
	Oranges or other citrus fruit	36.3%
	Other fruit juice	4.0%
**Flavones**	Cereal – wheat biscuits or wheat flakes (e.g., Weet Bix, Vita Brits, Weeties)	24.5%
	Herbal tea	16.0%
	Orange juice	9.8%
**Flavonols**	Tea	18.8%
	Dried beans, dried peas, chickpeas or lentils	14.9%
	Asian greens (e.g., bok choy)	9.7%
**Isoflavonoids**	Milk – soy	62.0%
	Bread – multigrain	29.7%
	Dried beans, dried peas, chickpeas or lentils	4.2%
**Hydroxybenzoic acids**	Tea	46.7%
	Spinach or silverbeet	9.8%
	Other nuts	9.0%
**Hydroxycinnamic acids**	Coffee	85.7%
	Dried beans, dried peas, chickpeas or lentils or lentils	1.7%
	Spinach or silverbeet	1.5%

Association between total polyphenols, and polyphenol classes and depressive symptoms: Sensitivity analyses

Table S9. Mean differences and 95% confidence intervals for the prospective association between energy-adjusted intake of total polyphenols and polyphenol classes and DASS depression subscale scores after excluding participants with a DASS depression subscale cut-off score of ≥ 14 at baseline

Polyphenol class	Quartile 1	Quartile 2	Quartile 3	Quartile 4	P for overall difference	Q value [a]
**Total polyphenols**						
Model 1	Referent	-0.63 (-1.34, 0.08)	-0.80 (-1.52, -0.08)	-0.37 (-1.12, 0.37)	0.14	0.50
Model 2	Referent	-0.53 (-1.53, 0.47)	0.03 (-0.99, 1.04)	-0.04 (-1.04, 0.96)	0.64	0.98
**Flavonoids**						
Model 1	Referent	-0.15 (-0.84, 0.55)	-0.09 (-0.80, 0.62)	-0.51 (-1.25, 0.22)	0.53	0.67
Model 2	Referent	-0.37 (-1.35, 0.61)	-0.32 (-1.31, 0.67)	-0.60 (-1.62, 0.41)	0.71	0.98
**Lignans**						
Model 1	Referent	0.15 (-0.55, 0.86)	-0.20 (-0.90, 0.51)	0.10 (-0.60, 0.81)	0.76	0.82
Model 2	Referent	-0.16 (-1.19, 0.86)	0.03 (-0.98, 1.04)	-0.11 (-1.08, 0.86)	0.98	0.98
**Other polyphenols**						
Model 1	Referent	-0.07 (-0.76, 0.63)	0.23 (-0.48, 0.95)	-0.31 (-1.03, 0.41)	0.47	0.67
Model 2	Referent	0.13 (-0.86, 1.12)	-0.15 (-1.14, 0.84)	0.08 (-0.91, 1.07)	0.94	0.98
**Phenolic acids**						
Model 1	Referent	-0.47 (-1.17, 0.24)	-0.48 (-1.20, 0.24)	-0.26 (-1.00, 0.48)	0.51	0.67
Model 2	Referent	-0.50 (-1.50, 0.50)	0.00 (-1.00, 1.01)	0.16 (-0.83, 1.16)	0.57	0.98
**Stilbenes**						
Model 1	Referent	0.29 (-0.42, 1.00)	0.38 (-0.33, 1.10)	0.60 (-0.14, 1.34)	0.46	0.67
Model 2	Referent	0.32 (-0.70, 1.35)	0.09 (-0.91, 1.10)	0.03 (-1.04, 1.10)	0.93	0.98

Model 1. Unadjusted for any covariate (N = 1,307)

Model 2. Adjusted for age, sex, education, employment, physical activity, alcohol intake, and smoking status (N = 1,007); reduced sample size due to missing covariate data

[a] Adjusted P for overall difference using the Simes method

Table S10. Mean differences and 95% confidence intervals for the prospective association between energy-adjusted intake of total polyphenols and polyphenol classes and DASS depression subscale scores after excluding participants with a self-diagnosis of depression at baseline

**Polyphenol class**	**Quartile 1**	**Quartile 2**	**Quartile 3**	**Quartile 4**	**P for overall difference**	**Q value [a]**
**Total polyphenols**						
Model 1	Referent	-0.36 (-1.10, 0.39)	-0.51 (-1.28, 0.27)	-0.40 (-1.21, 0.41)	0.62	0.94
Model 2	Referent	-0.48 (-1.55, 0.59)	0.06 (-1.03, 1.15)	-0.24 (-1.33, 0.84)	0.73	0.84
**Flavonoids**						
Model 1	Referent	-0.16 (-0.89, 0.58)	0.01 (-0.75, 0.76)	-0.21 (-1.00, 0.59)	0.92	0.99
Model 2	Referent	-0.41 (-1.46, 0.65)	-0.37 (-1.43, 0.69)	-0.45 (-1.55, 0.64)	0.84	0.84
**Lignans**						
Model 1	Referent	0.21 (-0.53, 0.96)	-0.51 (-1.27, 0.25)	-0.12 (-0.88, 0.64)	0.27	0.94
Model 2	Referent	0.24 (-0.84, 1.33)	-0.37 (-1.45, 0.71)	-0.35 (-1.40, 0.70)	0.63	0.84
**Other polyphenols**						
Model 1	Referent	-0.13 (-0.87, 0.61)	-0.12 (-0.88, 0.63)	-0.44 (-1.21, 0.33)	0.70	0.94
Model 2	Referent	0.06 (-1.01, 1.12)	-0.40 (-1.46, 0.66)	0.17 (-0.90, 1.24)	0.69	0.84
**Phenolic acids**						
Model 1	Referent	-0.39 (-1.13, 0.35)	-0.61 (-1.38, 0.16)	-0.53 (-1.33, 0.27)	0.45	0.94
Model 2	Referent	-0.75 (-1.82, 0.32)	-0.15 (-1.23, 0.93)	-0.38 (-1.46, 0.69)	0.53	0.84
**Stilbenes**						
Model 1	Referent	0.21 (-0.54, 0.97)	0.21 (-0.56, 0.98)	0.55 (-0.26, 1.36)	0.61	0.94
Model 2	Referent	0.43 (-0.68, 1.54)	-0.16 (-1.24, 0.92)	0.14 (-1.03, 1.31)	0.73	0.84

Model 1. Unadjusted for any covariate (N = 1,403)

Model 2. Adjusted for age, sex, education, employment, physical activity, alcohol intake, and smoking status (N = 1,078); reduced sample size due to missing covariate data

[a] Adjusted P for overall difference using the Simes method

Table S11. Mean differences and 95% confidence intervals for the prospective association between energy-adjusted intake of total polyphenols and polyphenol classes and DASS depression subscale scores after excluding participants with a self-diagnosis of anxiety at baseline

**Polyphenol class**	**Quartile 1**	**Quartile 2**	**Quartile 3**	**Quartile 4**	**P for overall difference**	**Q value [a]**
**Total polyphenols**						
Model 1	Referent	-0.46 (-1.22, 0.31)	-0.67 (-1.46, 0.12)	-0.45 (-1.27, 0.38)	0.41	0.95
Model 2	Referent	-0.69 (-1.78, 0.40)	-0.08 (-1.19, 1.03)	-0.44 (-1.54, 0.66)	0.55	0.80
**Flavonoids**						
Model 1	Referent	-0.01 (-0.77, 0.75)	0.18 (-0.59, 0.96)	0.05 (-0.76, 0.86)	0.95	0.95
Model 2	Referent	-0.35 (-1.43, 0.72)	-0.20 (-1.28, 0.88)	-0.35 (-1.46, 0.76)	0.91	0.91
**Lignans**						
Model 1	Referent	0.21 (-0.54, 0.97)	-0.47 (-1.25, 0.30)	-0.17 (-0.95, 0.60)	0.34	0.95
Model 2	Referent	0.29 (-0.82, 1.40)	-0.33 (-1.42, 0.77)	-0.35 (-1.42, 0.72)	0.63	0.80
**Other polyphenols**						
Model 1	Referent	-0.15 (-0.91, 0.60)	-0.15 (-0.93, 0.62)	-0.48 (-1.27, 0.31)	0.67	0.95
Model 2	Referent	0.08 (-1.00, 1.17)	-0.55 (-1.63, 0.53)	0.10 (-0.99, 1.19)	0.54	0.80
**Phenolic acids**						
Model 1	Referent	-0.49 (-1.25, 0.27)	-0.66 (-1.44, 0.13)	-0.65 (-1.47, 0.16)	0.35	0.95
Model 2	Referent	-0.81 (-1.91, 0.28)	-0.18 (-1.28, 0.91)	-0.50 (-1.60, 0.59)	0.46	0.80
**Stilbenes**						
Model 1	Referent	0.37 (-0.40, 1.14)	0.36 (-0.42, 1.14)	0.64 (-0.18, 1.46)	0.50	0.95
Model 2	Referent	0.37 (-0.76, 1.50)	-0.01 (-1.11, 1.08)	0.11 (-1.08, 1.29)	0.90	0.91

Model 1. Unadjusted for any covariate (N = 1,401)
Model 2. Adjusted for age, sex, education, employment, physical activity, alcohol intake, and smoking status (N = 1,073); reduced sample size due to missing covariate data
[a] Adjusted P for overall difference using the Simes method

Table S12. Mean differences and 95% confidence intervals for the prospective association between energy-adjusted intake of total polyphenols and polyphenol classes and DASS depression subscale scores after excluding participants with a body mass index of ≥ 30 kg/m^2 at baseline

Polyphenol class	Quartile 1	Quartile 2	Quartile 3	Quartile 4	P for overall difference	Q value [a]
**Total polyphenols**						
Model 1	Referent	-0.29 (-1.06, 0.49)	-0.55 (-1.35, 0.26)	-0.49 (-1.33, 0.35)	0.57	0.95
Model 2	Referent	-0.15 (-1.25, 0.94)	0.24 (-0.87, 1.36)	-0.19 (-1.31, 0.93)	0.84	0.94
**Flavonoids**						
Model 1	Referent	-0.04 (-0.80, 0.73)	0.32 (-0.47, 1.11)	0.11 (-0.71, 0.93)	0.79	0.95
Model 2	Referent	-0.27 (-1.36, 0.82)	-0.31 (-1.40, 0.78)	-0.12 (-1.22, 0.99)	0.94	0.94
**Lignans**						
Model 1	Referent	0.59 (-0.19, 1.36)	-0.32 (-1.10, 0.46)	0.16 (-0.63, 0.94)	0.12	0.56
Model 2	Referent	0.69 (-0.45, 1.82)	0.11 (-1.00, 1.22)	0.19 (-0.89, 1.26)	0.64	0.94
**Other polyphenols**						
Model 1	Referent	-0.16 (-0.93, 0.61)	0.00 (-0.78, 0.79)	-0.30 (-1.09, 0.50)	0.84	0.95
Model 2	Referent	0.41 (-0.69, 1.50)	-0.08 (-1.17, 1.01)	0.26 (-0.83, 1.35)	0.77	0.94
**Phenolic acids**						
Model 1	Referent	-0.40 (-1.16, 0.37)	-0.63 (-1.43, 0.16)	-0.79 (-1.62, 0.04)	0.28	0.79
Model 2	Referent	-0.41 (-1.52, 0.69)	-0.02 (-1.13, 1.08)	-0.41 (-1.52, 0.71)	0.78	0.94
**Stilbenes**						
Model 1	Referent	0.42 (-0.37, 1.21)	0.63 (-0.17, 1.43)	0.93 (0.10, 1.77)	0.17	0.60
Model 2	Referent	0.69 (-0.47, 1.85)	0.31 (-0.81, 1.43)	0.64 (-0.56, 1.84)	0.61	0.94

Model 1. Unadjusted for any covariate (N = 1,363)

Model 2. Adjusted for age, sex, education, employment, physical activity, alcohol intake, and smoking status (N = 1,044); reduced sample size due to missing covariate data

[a] Adjusted P for overall difference using the Simes method

Table S13. Mean differences and 95% confidence intervals for the prospective association between energy-unadjusted intake of total polyphenols and polyphenol classes and DASS depression subscale scores

Polyphenol class	Quartile 1	Quartile 2	Quartile 3	Quartile 4	P for overall difference	Q value [a]
**Total polyphenols**						
Median (IQR) (mg/day)	290.69 (215.43, 378.09)	583.18 (474.28, 729.98)	986.05 (809.95, 1172.25)	1737.96 (1498.94, 2049.46)		
Model 1	Referent	-0.49 (-1.24, 0.26)	-0.37 (-1.16, 0.42)	-0.14 (-0.97, 0.69)	0.58	0.68
Model 2	Referent	-0.93 (-2.00, 0.14)	-0.15 (-1.25, 0.94)	-0.35 (-1.46, 0.76)	0.32	0.47
**Flavonoids**						
Median (IQR) (mg/day)	143.68 (111.80, 171.45)	252.01 (220.68, 280.49)	383.45 (345.76, 433.20)	654.16 (563.33, 824.64)		
Model 1	Referent	-0.60 (-1.35, 0.14)	-0.49 (-1.26, 0.29)	-0.29 (-1.10, 0.52)	0.42	0.59
Model 2	Referent	-1.03 (-2.07, 0.02)	-1.14 (-2.21, -0.07)	-0.53 (-1.62, 0.56)	0.13	0.31
**Lignans**						
Median (IQR) (mg/day)	0.58 (0.46, 0.67)	1.01 (0.88, 1.17)	2.00 (1.52, 2.68)	8.25 (5.20, 11.79)		
Model 1	Referent	-1.11 (-1.87, -0.35)	-1.27 (-2.06, -0.49)	-0.61 (-1.40, 0.18)	0.006	0.03
Model 2	Referent	-1.26 (-2.34, -0.18)	-1.71 (-2.80, -0.63)	-1.03 (-2.13, 0.06)	0.02	0.08
**Other polyphenols**						
Median (IQR) (mg/day)	9.80 (6.70, 12.75)	20.81 (17.49, 24.17)	33.78 (30.03, 37.79)	58.14 (48.33, 75.18)		
Model 1	Referent	-0.15 (-0.91, 0.60)	-0.39 (-1.17, 0.38)	-0.27 (-1.07, 0.53)	0.79	0.79
Model 2	Referent	-0.80 (-1.86, 0.26)	-0.51 (-1.58, 0.55)	-0.19 (-1.30, 0.92)	0.46	0.55
**Phenolic acids**						
Median (IQR) (mg/day)	71.30 (51.08, 98.54)	180.88 (126.97, 315.56)	514.73 (339.33, 603.71)	1258.14 (870.03, 1370.64)		
Model 1	Referent	-1.07 (-1.82, -0.32)	-0.80 (-1.60, -0.01)	-0.86 (-1.69, -0.03)	0.04	0.10
Model 2	Referent	-1.05 (-2.13, 0.02)	-0.17 (-1.29, 0.95)	-0.55 (-1.67, 0.57)	0.21	0.41
**Stilbenes**						
Median (IQR) (mg/day)	0.03 (0.02, 0.05)	0.15 (0.10, 0.21)	0.56 (0.40, 0.76)	2.52 (1.66, 4.19)		
Model 1	Referent	-0.82 (-1.58, -0.06)	0.47 (-0.32, 1.26)	0.32 (-0.52, 1.15)	0.004	0.03
Model 2	Referent	-0.91 (-2.00, 0.17)	-0.12 (-1.22, 0.98)	-0.33 (-1.54, 0.88)	0.33	0.47

IQR, interquartile range
Model 1. Unadjusted for any covariate (N = 1,484)
Model 2. Adjusted for age, sex, education, employment, physical activity, smoking status, and alcohol intake (N = 1,137); reduced sample size due to missing covariate data
[a] Adjusted P for overall difference using the Simes method

Association between total polyphenols, and polyphenol classes and depressive symptoms: Exploratory analyses

Table S14. Odds ratios (OR) and 95% confidence intervals (CI) for the prospective association between energy-adjusted intake of total polyphenols and polyphenol classes and depression incidence

Polyphenol class	Quartile 1	Quartile 2	Quartile 3	Quartile 4	P for overall difference	Q value [a]
**Total polyphenols**						
Model 1 [OR (95% CI)]	Referent	0.82 (0.57, 1.20)	0.87 (0.59, 1.27)	0.81 (0.54, 1.19)	0.69	0.82
Model 2 [OR (95% CI)]	Referent	0.89 (0.52, 1.53)	1.22 (0.72, 2.08)	0.96 (0.56, 1.64)	0.65	0.79
**Flavonoids**						
Model 1 [OR (95% CI)]	Referent	0.92 (0.63, 1.32)	1.05 (0.72, 1.52)	0.90 (0.61, 1.33)	0.82	0.82
Model 2 [OR (95% CI)]	Referent	0.82 (0.49, 1.40)	0.89 (0.53, 1.50)	0.85 (0.50, 1.45)	0.90	0.93
**Lignans**						
Model 1 [OR (95% CI)]	Referent	0.97 (0.67, 1.40)	0.73 (0.50, 1.06)	0.88 (0.61, 1.28)	0.35	0.82
Model 2 [OR (95% CI)]	Referent	0.82 (0.48, 1.40)	0.63 (0.37, 1.07)	0.72 (0.43, 1.21)	0.37	0.79
**Other polyphenols**						
Model 1 [OR (95% CI)]	Referent	0.87 (0.61, 1.26)	0.93 (0.64, 1.35)	0.80 (0.55, 1.18)	0.70	0.82
Model 2 [OR (95% CI)]	Referent	1.12 (0.65, 1.91)	0.97 (0.57, 1.65)	1.10 (0.64, 1.89)	0.93	0.93
**Phenolic acids**						
Model 1 [OR (95% CI)]	Referent	0.81 (0.56, 1.18)	0.89 (0.61, 1.30)	0.75 (0.51, 1.12)	0.50	0.82
Model 2 [OR (95% CI)]	Referent	0.78 (0.45, 1.34)	1.07 (0.63, 1.83)	0.92 (0.54, 1.57)	0.67	0.79
**Stilbenes**						
Model 1 [OR (95% CI)]	Referent	1.14 (0.78, 1.66)	1.17 (0.80, 1.72)	1.22 (0.82, 1.81)	0.78	0.82
Model 2 [OR (95% CI)]	Referent	1.35 (0.77, 2.37)	1.13 (0.65, 1.95)	1.32 (0.75, 2.34)	0.68	0.79

Model 1. Unadjusted for any covariate (N = 1,484)

Model 2. Adjusted for age, sex, education, employment, physical activity, smoking status, and alcohol intake (N = 1,137); reduced sample size due to missing covariate data

[a] Adjusted P for overall difference using the Simes method

Table S15. Mean differences and 95% confidence intervals for the prospective association between energy-adjusted intake of total polyphenols and polyphenol classes and total DASS score

Polyphenol class	Quartile 1	Quartile 2	Quartile 3	Quartile 4	P for overall difference	Q value [a]
**Total polyphenols**						
Model 1	Referent	-0.61 (-2.45, 1.22)	-0.41 (-2.32, 1.51)	0.64 (-1.37, 2.65)	0.56	0.94
Model 2	Referent	-1.46 (-4.08, 1.17)	0.26 (-2.41, 2.94)	0.44 (-2.25, 3.13)	0.45	0.97
**Flavonoids**						
Model 1	Referent	0.48 (-1.33, 2.29)	0.78 (-1.08, 2.64)	0.87 (-1.10, 2.83)	0.82	0.94
Model 2	Referent	-0.17 (-2.76, 2.41)	-0.72 (-3.33, 1.89)	-0.11 (-2.81, 2.59)	0.95	0.97
**Lignans**						
Model 1	Referent	1.04 (-0.79, 2.86)	-0.75 (-2.61, 1.11)	-0.30 (-2.17, 1.57)	0.25	0.70
Model 2	Referent	0.02 (-2.66, 2.70)	-1.22 (-3.88, 1.43)	-1.19 (-3.79, 1.40)	0.66	0.97
**Other polyphenols**						
Model 1	Referent	-0.04 (-1.86, 1.77)	0.50 (-1.36, 2.37)	-0.25 (-2.15, 1.66)	0.86	0.94
Model 2	Referent	0.34 (-2.29, 2.96)	0.00 (-2.61, 2.61)	0.96 (-1.68, 3.61)	0.86	0.97
**Phenolic acids**						
Model 1	Referent	-0.48 (-2.30, 1.34)	-0.35 (-2.24, 1.55)	0.36 (-1.63, 2.35)	0.80	0.94
Model 2	Referent	-1.10 (-3.73, 1.53)	-0.06 (-2.71, 2.60)	0.59 (-2.08, 3.26)	0.63	0.97
**Stilbenes**						
Model 1	Referent	0.71 (-1.15, 2.56)	2.11 (0.21, 4.00)	2.43 (0.41, 4.44)	0.05	0.26
Model 2	Referent	0.63 (-2.10, 3.36)	0.20 (-2.46, 2.86)	0.42 (-2.46, 3.31)	0.97	0.97

Model 1. Unadjusted for any covariate (N = 1,483)

Model 2. Adjusted for age, sex, education, employment, physical activity, alcohol intake, and smoking status (N = 1,135); reduced sample size due to missing covariate data

[a] Adjusted P for overall difference using the Simes method

Table S16. Mean differences and 95% confidence intervals for the prospective association between energy-adjusted intake of total polyphenols and polyphenol classes and DASS depression subscale scores after excluding polyphenols from alcohol

Polyphenol class	Quartile 1	Quartile 2	Quartile 3	Quartile 4	P for overall difference	Q value [a]
**Total polyphenols**						
Energy-adjusted (mg/day) [b] Median (IQR)	-566.90 (-668.24, -502.52)	-314.50 (-376.77, -239.36)	26.38 (-82.68, 168.64)	667.49 (509.36, 1020.57)		
Energy-unadjusted (mg/day) [c] Median (IQR)	293.34 (206.41, 424.89)	543.64 (422.41, 716.17)	926.95 (752.06, 1145.18)	1670.36 (1398.46, 1984.14)		
Model 1	Referent	-0.13 (-0.89, 0.63)	-0.60 (-1.38, 0.19)	-0.46 (-1.28, 0.37)	0.43	0.79
Model 2	Referent	-0.30 (-1.38, 0.78)	-0.22 (-1.31, 0.87)	-0.15 (-1.26, 0.95)	0.96	0.96
**Flavonoids**						
Energy-adjusted (mg/day) [b] Median (IQR)	-208.44 (-245.16, -183.84)	-112.49 (-135.27, -89.51)	-2.35 (-35.04, 41.68)	247.35 (163.65, 411.57)		
Energy-unadjusted (mg/day) [c] Median (IQR)	139.86 (104.30, 183.73)	224.34 (189.59, 271.22)	352.31 (303.79, 404.88)	617.71 (524.02, 795.88)		
Model 1	Referent	-0.05 (-0.80, 0.71)	0.02 (-0.75, 0.80)	-0.19 (-1.01, 0.62)	0.95	0.95
Model 2	Referent	0.21 (-0.86, 1.28)	-0.22 (-1.30, 0.86)	-0.21 (-1.32, 0.91)	0.84	0.93
**Lignans**						
Energy-adjusted (mg/day) [b] Median (IQR)	-2.75 (-3.11, -2.51)	-2.03 (-2.21, -1.79)	-1.11 (-1.61, -0.34)	5.08 (2.02, 7.97)		
Energy-unadjusted (mg/day) [c] Median (IQR)	0.76 (0.55, 1.07)	0.80 (0.58, 1.18)	1.67 (1.05, 2.49)	8.21 (5.06, 11.74)		
Model 1	Referent	0.08 (-0.68, 0.83)	-0.42 (-1.19, 0.36)	-0.03 (-0.81, 0.74)	0.57	0.82
Model 2	Referent	0.41 (-0.69, 1.51)	-0.29 (-1.39, 0.82)	-0.04 (-1.11, 1.03)	0.65	0.93

Polyphenol class	Quartile 1	Quartile 2	Quartile 3	Quartile 4	P for overall difference	Q value [a]
**Other polyphenols**						
Energy-adjusted (mg/day) [b] Median (IQR)	-20.31 (-25.30, -16.97)	-9.55 (-11.84, -7.20)	0.22 (-2.54, 3.76)	20.62 (12.94, 37.46)		
Energy-unadjusted (mg/day) [c] Median (IQR)	11.01 (7.20, 17.03)	18.96 (12.97, 25.01)	29.82 (23.21, 35.92)	54.39 (43.97, 71.57)		
Model 1	Referent	-0.13 (-0.89, 0.63)	-0.09 (-0.86, 0.68)	-0.58 (-1.37, 0.21)	0.45	0.79
Model 2	Referent	0.53 (-0.54, 1.61)	0.15 (-0.93, 1.22)	0.32 (-0.77, 1.41)	0.78	0.93
**Phenolic acids**						
Energy-adjusted (mg/day) [b] Median (IQR)	-427.86 (-502.49, -342.39)	-277.44 (-340.99, -240.05)	-46.75 (-111.67, 42.35)	629.35 (426.85, 778.72)		
Energy-unadjusted (mg/day) [c] Median (IQR)	76.76 (50.22, 119.04)	168.85 (106.28, 298.56)	491.80 (321.61, 589.62)	1227.98 (860.72, 1350.83)		
Model 1	Referent	-0.46 (-1.22, 0.31)	-0.70 (-1.49, 0.09)	-0.81 (-1.63, 0.02)	0.23	0.55
Model 2	Referent	-0.79 (-1.88, 0.30)	-0.09 (-1.18, 1.00)	-0.50 (-1.59, 0.59)	0.44	0.93
**Stilbenes**						
Energy-adjusted (mg/day) [b] Median (IQR)	-0.05 (-0.05, -0.04)	-0.03 (-0.03, -0.02)	0.00 (-0.01, 0.01)	0.05 (0.03, 0.10)		
Energy-unadjusted (mg/day) [c] Median (IQR)	0.02 (0.01, 0.03)	0.04 (0.03, 0.05)	0.06 (0.05, 0.08)	0.12 (0.10, 0.17)		
Model 1	Referent	-0.27 (-1.02, 0.48)	-0.75 (-1.52, 0.02)	-0.70 (-1.48, 0.09)	0.19	0.54
Model 2	Referent	-0.67 (-1.74, 0.40)	-0.79 (-1.88, 0.29)	-0.71 (-1.81, 0.39)	0.47	0.93

IQR, interquartile range

Model 1. Unadjusted for any covariate (N = 1,484)

Model 2. Adjusted for age, sex, education, employment, physical activity, smoking status, and alcohol intake (N = 1,137); reduced sample size due to missing covariate data

[a] Adjusted P for overall difference using the Simes method

[b] Energy-adjusted values derived using Willett's residual method

[c] Energy-unadjusted values reflect intakes within energy-adjusted polyphenol intake quartiles

Association between total polyphenols, and polyphenol classes and depressive symptoms: Subgroup analyses

Table S17. Mean differences and 95% confidence intervals for the prospective association between energy-adjusted intake of total polyphenols and polyphenol classes and DASS depression subscale scores by smoking status at baseline

Polyphenol class	Smoking status	Quartile 1	Quartile 2	Quartile 3	Quartile 4	P for overall difference	Q value [a]
**Total polyphenols**	Non-smoker	Referent	-0.24 (-1.34, 0.86)	-0.84 (-2.00, 0.33)	-0.48 (-1.69, 0.73)	0.54	0.84
	Current smoker	Referent	-2.00 (-5.29, 1.30)	1.92 (-1.54, 5.39)	0.84 (-2.47, 4.15)	0.16	0.44
**Flavonoids**	Non-smoker	Referent	0.27 (-0.88, 1.42)	0.02 (-1.12, 1.16)	-0.35 (-1.57, 0.87)	0.75	0.84
	Current smoker	Referent	-1.04 (-3.95, 1.87)	1.29 (-2.10, 4.68)	3.92 (0.81, 7.03)	0.03	0.17
**Lignans**	Non-smoker	Referent	1.03 (-0.11, 2.18)	0.12 (-1.03, 1.28)	0.71 (-0.45, 1.88)	0.20	0.72
	Current smoker	Referent	0.61 (-2.58, 3.80)	-0.47 (-3.61, 2.67)	-0.85 (-3.75, 2.06)	0.85	0.99
**Other polyphenols**	Non-smoker	Referent	0.73 (-0.39, 1.86)	-0.41 (-1.54, 0.71)	-0.22 (-1.35, 0.90)	0.17	0.72
	Current smoker	Referent	0.46 (-2.78, 3.70)	-0.01 (-3.31, 3.29)	1.44 (-2.15, 5.04)	0.82	0.99
**Phenolic acids**	Non-smoker	Referent	-0.44 (-1.52, 0.65)	-0.62 (-1.77, 0.53)	-0.68 (-1.88, 0.51)	0.68	0.84
	Current smoker	Referent	-0.23 (-3.57, 3.11)	0.22 (-3.23, 3.66)	0.30 (-3.09, 3.70)	0.99	0.99
**Stilbenes**	Non-smoker	Referent	0.14 (-1.02, 1.30)	-0.02 (-1.21, 1.16)	-0.44 (-1.81, 0.94)	0.84	0.84
	Current smoker	Referent	-1.11 (-4.77, 2.55)	-1.44 (-4.75, 1.87)	-0.22 (-3.44, 3.00)	0.81	0.99

Adjusted for age, sex, education, employment, physical activity, and alcohol intake

Non-smoker (N = 737)

Current smoker (N = 110)

[a] Adjusted P for overall difference using the Simes method

Table S18. Mean differences and 95% confidence intervals for the prospective association between energy-adjusted intake of total polyphenols and polyphenol classes and DASS depression subscale scores by physical activity status at baseline

Polyphenol class	Physical activity guidelines	Quartile 1	Quartile 2	Quartile 3	Quartile 4	P for overall difference	Q value [a]
**Total polyphenols**	Not met	Referent	-0.73 (-3.09, 1.62)	-1.72 (-4.20, 0.76)	-0.87 (-3.47, 1.72)	0.60	0.71
	Met	Referent	0.01 (-1.35, 1.38)	0.62 (-0.82, 2.06)	0.01 (-1.43, 1.45)	0.76	0.89
**Flavonoids**	Not met	Referent	-0.20 (-2.66, 2.26)	-1.78 (-4.21, 0.65)	0.15 (-2.41, 2.71)	0.32	0.65
	Met	Referent	0.51 (-0.84, 1.86)	1.15 (-0.22, 2.53)	0.50 (-0.96, 1.95)	0.42	0.65
**Lignans**	Not met	Referent	-0.93 (-3.25, 1.39)	-1.94 (-4.37, 0.50)	-1.36 (-3.88, 1.16)	0.47	0.65
	Met	Referent	1.39 (-0.01, 2.80)	0.59 (-0.84, 2.03)	0.75 (-0.63, 2.12)	0.27	0.64
**Other polyphenols**	Not met	Referent	0.28 (-2.14, 2.70)	-1.21 (-3.68, 1.26)	-0.60 (-3.14, 1.93)	0.61	0.71
	Met	Referent	1.06 (-0.31, 2.43)	0.12 (-1.25, 1.48)	0.89 (-0.49, 2.27)	0.27	0.64
**Phenolic acids**	Not met	Referent	0.03 (-2.28, 2.35)	-2.24 (-4.73, 0.25)	-0.86 (-3.47, 1.76)	0.23	0.65
	Met	Referent	-0.20 (-1.57, 1.18)	0.50 (-0.91, 1.90)	-0.18 (-1.59, 1.23)	0.71	0.89
**Stilbenes**	Not met	Referent	-1.22 (-3.74, 1.31)	-0.79 (-3.29, 1.72)	-2.25 (-5.01, 0.50)	0.42	0.65
	Met	Referent	1.37 (-0.05, 2.79)	0.46 (-0.93, 1.85)	1.26 (-0.33, 2.85)	0.18	0.64

Adjusted for age, sex, education, employment, smoking status, and alcohol intake
Not met (N = 223)
Met (N = 600)
[a] Adjusted P for overall difference using the Simes method

Table S19. Mean differences and 95% confidence intervals for the prospective association between energy-adjusted intake of total polyphenols and polyphenol classes and DASS depression subscale scores by alcohol consumption at baseline

**Polyphenol class**	**Alcohol consumption**	**Quartile 1**	**Quartile 2**	**Quartile 3**	**Quartile 4**	**P for overall difference**	**Q value [a]**
**Total polyphenols**	Nil to low	Referent	-0.52 (-2.00, 0.96)	-0.35 (-1.93, 1.23)	-0.82 (-2.46, 0.82)	0.79	0.94
	Moderate to high	Referent	0.09 (-2.24, 2.43)	1.55 (-0.74, 3.85)	1.41 (-0.79, 3.60)	0.41	0.56
**Flavonoids**	Nil to low	Referent	0.15 (-1.37, 1.68)	-1.10 (-2.65, 0.44)	-0.56 (-2.21, 1.10)	0.32	0.63
	Moderate to high	Referent	0.20 (-1.94, 2.34)	2.85 (0.72, 4.98)	1.95 (-0.20, 4.10)	0.03	0.22
**Lignans**	Nil to low	Referent	-0.03 (-1.57, 1.52)	-0.93 (-2.50, 0.64)	-0.62 (-2.22, 0.98)	0.57	0.80
	Moderate to high	Referent	2.12 (-0.11, 4.36)	2.00 (-0.18, 4.17)	1.95 (-0.07, 3.96)	0.15	0.35
**Other polyphenols**	Nil to low	Referent	1.66 (0.13, 3.18)	-0.49 (-1.99, 1.02)	-0.31 (-1.86, 1.25)	0.01	0.18
	Moderate to high	Referent	0.88 (-1.33, 3.09)	1.46 (-0.72, 3.64)	2.68 (0.47, 4.89)	0.11	0.35
**Phenolic acids**	Nil to low	Referent	-0.35 (-1.81, 1.12)	-0.43 (-1.99, 1.13)	-0.46 (-2.07, 1.16)	0.94	0.94
	Moderate to high	Referent	-0.28 (-2.62, 2.07)	1.22 (-1.04, 3.48)	0.73 (-1.41, 2.88)	0.56	0.61
**Stilbenes**	Nil to low	Referent	-0.42 (-1.92, 1.09)	-0.60 (-2.11, 0.92)	-0.54 (-2.31, 1.23)	0.88	0.94
	Moderate to high	Referent	2.19 (-0.52, 4.90)	2.64 (0.24, 5.04)	3.78 (1.43, 6.13)	0.02	0.22

Adjusted for age, sex, education, employment, physical activity, and smoking status
Nil to low (N = 524)
Moderate to high (N = 242)
[a] Adjusted P for overall difference using the Simes method

Table S20. Mean differences and 95% confidence intervals for the prospective association between energy-adjusted intake of total polyphenols and polyphenol classes and DASS depression subscale scores by sex

**Polyphenol class**	**Sex**	**Quartile 1**	**Quartile 2**	**Quartile 3**	**Quartile 4**	**P for overall difference**	**Q value [a]**
**Total polyphenols**	Male	Referent	0.45 (-1.01, 1.91)	1.01 (-0.45, 2.47)	0.48 (-0.99, 1.96)	0.61	0.71
	Female	Referent	-1.91 (-3.51, -0.32)	-1.50 (-3.16, 0.15)	-1.56 (-3.22, 0.10)	0.12	0.41
**Flavonoids**	Male	Referent	0.05 (-1.37, 1.47)	0.78 (-0.62, 2.18)	0.36 (-1.14, 1.87)	0.70	0.76
	Female	Referent	-0.40 (-2.03, 1.23)	-1.24 (-2.91, 0.43)	-1.29 (-2.96, 0.39)	0.33	0.47
**Lignans**	Male	Referent	0.08 (-1.35, 1.50)	0.02 (-1.58, 1.61)	0.37 (-0.96, 1.70)	0.95	0.95
	Female	Referent	-0.19 (-2.01, 1.62)	-1.03 (-2.74, 0.68)	-0.77 (-2.59, 1.06)	0.54	0.68
**Other polyphenols**	Male	Referent	0.32 (-1.14, 1.78)	-0.47 (-1.94, 1.01)	0.95 (-0.42, 2.33)	0.27	0.69
	Female	Referent	0.15 (-1.47, 1.77)	-0.37 (-1.98, 1.24)	-0.69 (-2.43, 1.04)	0.69	0.75
**Phenolic acids**	Male	Referent	0.23 (-1.25, 1.71)	0.98 (-0.49, 2.45)	-0.04 (-1.50, 1.42)	0.48	0.69
	Female	Referent	-1.79 (-3.37, -0.21)	-1.30 (-2.90, 0.31)	-0.98 (-2.62, 0.66)	0.16	0.41
**Stilbenes**	Male	Referent	2.00 (0.54, 3.47)	0.76 (-0.66, 2.18)	1.41 (-0.11, 2.92)	0.04	0.47
	Female	Referent	-1.40 (-3.24, 0.44)	-1.31 (-3.11, 0.50)	-1.97 (-3.94, 0.00)	0.26	0.41

Adjusted for age, education, employment, physical activity, smoking status, and alcohol intake
Male (N = 541)
Female (N = 596)
[a] Adjusted P for overall difference using the Simes method

Association between polyphenol subclasses and depressive symptoms: Sensitivity analyses

Table S21. Mean differences and 95% confidence intervals for the prospective association between energy-adjusted intake of polyphenol subclasses and DASS depression subscale scores after excluding participants with a DASS depression subscale cut-off score of ≥ 14 at baseline

Polyphenol subclass	Quartile 1	Quartile 2	Quartile 3	Quartile 4	P for overall difference	Q value [a]
**Anthocyanins**						
Model 1	Referent	-0.50 (-1.20, 0.20)	-0.44 (-1.14, 0.27)	-0.48 (-1.20, 0.24)	0.47	0.67
Model 2	Referent	-0.54 (-1.53, 0.45)	-0.85 (-1.83, 0.13)	-0.36 (-1.34, 0.63)	0.38	0.98
**Flavanols**						
Model 1	Referent	-0.16 (-0.85, 0.54)	0.01 (-0.70, 0.73)	-0.11 (-0.85, 0.62)	0.95	0.95
Model 2	Referent	-0.43 (-1.41, 0.55)	-0.22 (-1.20, 0.77)	-0.36 (-1.37, 0.66)	0.84	0.98
**Flavanones**						
Model 1	Referent	0.28 (-0.40, 0.96)	0.11 (-0.58, 0.79)	-0.54 (-1.24, 0.16)	0.12	0.50
Model 2	Referent	0.49 (-0.46, 1.44)	0.56 (-0.38, 1.51)	-0.04 (-1.00, 0.92)	0.47	0.98
**Flavones**						
Model 1	Referent	-0.02 (-0.70, 0.67)	0.11 (-0.57, 0.80)	-0.44 (-1.14, 0.26)	0.42	0.67
Model 2	Referent	-0.07 (-1.04, 0.91)	0.35 (-0.63, 1.33)	-0.42 (-1.40, 0.55)	0.46	0.98
**Flavonols**						
Model 1	Referent	-0.76 (-1.45, -0.07)	-0.88 (-1.60, -0.17)	-0.93 (-1.66, -0.19)	0.04	0.38
Model 2	Referent	-1.32 (-2.29, -0.36)	-1.12 (-2.11, -0.12)	-1.32 (-2.32, -0.32)	0.03	0.20
**Isoflavonoids**						
Model 1	Referent	-0.50 (-1.17, 0.17)	-0.52 (-1.22, 0.17)	0.28 (-0.44, 0.99)	0.05	0.38
Model 2	Referent	-1.10 (-2.06, -0.13)	-0.90 (-1.87, 0.06)	0.03 (-0.96, 1.02)	0.03	0.20
**Hydroxybenzoic acids**						
Model 1	Referent	0.02 (-0.68, 0.73)	0.11 (-0.61, 0.83)	-0.32 (-1.07, 0.43)	0.66	0.77
Model 2	Referent	-0.70 (-1.70, 0.31)	-0.95 (-1.97, 0.07)	-0.97 (-2.01, 0.07)	0.23	0.98
**Hydroxycinnamic acids**						
Model 1	Referent	-0.54 (-1.23, 0.16)	-0.48 (-1.20, 0.25)	-0.12 (-0.86, 0.62)	0.35	0.67
Model 2	Referent	-0.10 (-1.11, 0.91)	0.04 (-0.99, 1.07)	0.51 (-0.52, 1.54)	0.61	0.98

Model 1. Unadjusted for any covariate (N = 1,307)

Model 2. Adjusted for age, education, employment, physical activity, smoking status, and alcohol intake (N = 1,007); reduced sample size due to missing covariate data

[a] Adjusted P for overall difference using the Simes method

Table S22. Mean differences and 95% confidence intervals for the prospective association between energy-adjusted intake of polyphenol subclasses and DASS depression subscale scores after excluding participants with a self-diagnosis of depression at baseline

Polyphenol subclass	Quartile 1	Quartile 2	Quartile 3	Quartile 4	P for overall difference	Q value [a]
**Anthocyanins**						
Model 1	Referent	-0.34 (-1.08, 0.40)	-0.38 (-1.13, 0.37)	-0.45 (-1.22, 0.33)	0.67	0.94
Model 2	Referent	-0.78 (-1.84, 0.28)	-0.76 (-1.80, 0.29)	-0.39 (-1.46, 0.67)	0.43	0.84
**Flavanols**						
Model 1	Referent	-0.07 (-0.81, 0.66)	-0.07 (-0.83, 0.69)	0.01 (-0.78, 0.81)	0.99	0.99
Model 2	Referent	-0.68 (-1.73, 0.37)	-0.53 (-1.59, 0.53)	-0.51 (-1.60, 0.59)	0.62	0.84
**Flavanones**						
Model 1	Referent	0.31 (-0.41, 1.03)	0.09 (-0.65, 0.83)	-0.09 (-0.84, 0.66)	0.74	0.94
Model 2	Referent	0.32 (-0.70, 1.34)	0.46 (-0.57, 1.49)	0.55 (-0.48, 1.57)	0.74	0.84
**Flavones**						
Model 1	Referent	0.17 (-0.55, 0.89)	0.06 (-0.67, 0.79)	-0.18 (-0.93, 0.56)	0.82	0.96
Model 2	Referent	-0.07 (-1.10, 0.96)	0.25 (-0.79, 1.29)	-0.28 (-1.33, 0.77)	0.78	0.84
**Flavonols**						
Model 1	Referent	-0.81 (-1.54, -0.08)	-0.54 (-1.30, 0.22)	-0.68 (-1.47, 0.11)	0.16	0.76
Model 2	Referent	-1.73 (-2.77, -0.69)	-1.06 (-2.13, 0.01)	-1.45 (-2.54, -0.37)	0.008	0.11
**Isoflavonoids**						
Model 1	Referent	-0.60 (-1.32, 0.11)	-0.71 (-1.44, 0.03)	0.24 (-0.52, 1.00)	0.02	0.33
Model 2	Referent	-1.16 (-2.20, -0.11)	-1.13 (-2.17, -0.09)	-0.08 (-1.14, 0.98)	0.03	0.19
**Hydroxybenzoic acids**						
Model 1	Referent	-0.19 (-0.93, 0.55)	0.16 (-0.61, 0.93)	-0.21 (-1.02, 0.59)	0.71	0.94
Model 2	Referent	-1.02 (-2.10, 0.06)	-1.34 (-2.44, -0.24)	-1.21 (-2.33, -0.09)	0.08	0.40
**Hydroxycinnamic acids**						
Model 1	Referent	-0.82 (-1.55, -0.09)	-0.77 (-1.54, 0.00)	-0.43 (-1.23, 0.38)	0.11	0.76
Model 2	Referent	-0.60 (-1.68, 0.48)	-0.41 (-1.51, 0.69)	-0.03 (-1.14, 1.09)	0.61	0.84

Model 1. Unadjusted for any covariate (N = 1,403)

Model 2. Adjusted for age, education, employment, physical activity, smoking status, and alcohol intake (N = 1,078); reduced sample size due to missing covariate data

[a] Adjusted P for overall difference using the Simes method

Table S23. Mean differences and 95% confidence intervals for the prospective association between energy-adjusted intake of polyphenol subclasses and DASS depression subscale scores after excluding participants with a self-diagnosis of anxiety at baseline

Polyphenol subclass	Quartile 1	Quartile 2	Quartile 3	Quartile 4	P for overall difference	Q value [a]
**Anthocyanins**						
Model 1	Referent	-0.30 (-1.05, 0.45)	-0.35 (-1.11, 0.42)	-0.29 (-1.08, 0.50)	0.81	0.95
Model 2	Referent	-0.69 (-1.78, 0.39)	-0.65 (-1.72, 0.43)	-0.34 (-1.43, 0.75)	0.57	0.80
**Flavanols**						
Model 1	Referent	0.01 (-0.74, 0.77)	0.11 (-0.67, 0.89)	0.26 (-0.55, 1.07)	0.92	0.95
Model 2	Referent	-0.72 (-1.79, 0.35)	-0.45 (-1.52, 0.63)	-0.48 (-1.59, 0.63)	0.62	0.80
**Flavanones**						
Model 1	Referent	0.36 (-0.37, 1.10)	0.16 (-0.60, 0.91)	0.06 (-0.70, 0.83)	0.79	0.95
Model 2	Referent	0.59 (-0.45, 1.64)	0.76 (-0.29, 1.82)	0.78 (-0.27, 1.83)	0.43	0.80
**Flavones**						
Model 1	Referent	0.35 (-0.38, 1.08)	0.09 (-0.66, 0.84)	-0.06 (-0.82, 0.71)	0.71	0.95
Model 2	Referent	0.01 (-1.04, 1.06)	0.38 (-0.69, 1.44)	-0.06 (-1.13, 1.01)	0.84	0.91
**Flavonols**						
Model 1	Referent	-0.68 (-1.42, 0.07)	-0.48 (-1.26, 0.30)	-0.50 (-1.30, 0.31)	0.35	0.95
Model 2	Referent	-1.79 (-2.86, -0.73)	-1.15 (-2.24, -0.05)	-1.37 (-2.47, -0.27)	0.009	0.13
**Isoflavonoids**						
Model 1	Referent	-0.55 (-1.28, 0.17)	-0.69 (-1.44, 0.06)	0.20 (-0.58, 0.97)	0.05	0.46
Model 2	Referent	-1.14 (-2.21, -0.08)	-1.19 (-2.24, -0.14)	-0.13 (-1.22, 0.95)	0.03	0.23
**Hydroxybenzoic acids**						
Model 1	Referent	-0.35 (-1.11, 0.41)	0.03 (-0.76, 0.82)	-0.24 (-1.06, 0.58)	0.70	0.95
Model 2	Referent	-1.30 (-2.40, -0.19)	-1.44 (-2.57, -0.32)	-1.23 (-2.37, -0.09)	0.05	0.25
**Hydroxycinnamic acids**						
Model 1	Referent	-0.98 (-1.73, -0.23)	-0.81 (-1.60, -0.02)	-0.56 (-1.38, 0.26)	0.07	0.46
Model 2	Referent	-0.74 (-1.85, 0.38)	-0.41 (-1.53, 0.72)	-0.18 (-1.31, 0.96)	0.58	0.80

Model 1. Unadjusted for any covariate (N = 1,401)
Model 2. Adjusted for age, education, employment, physical activity, smoking status, and alcohol intake (N = 1,073); reduced sample size due to missing covariate data
[a] Adjusted P for overall difference using the Simes method

Table S24. Mean differences and 95% confidence intervals for the prospective association between energy-adjusted intake of polyphenol subclasses and DASS depression subscale scores after excluding participants with a body mass index of ≥ 30 kg/m^2 at baseline

Polyphenol subclass	Quartile 1	Quartile 2	Quartile 3	Quartile 4	P for overall difference	Q value [a]
**Anthocyanins**						
Model 1	Referent	-0.38 (-1.15, 0.38)	-0.39 (-1.17, 0.38)	-0.33 (-1.13, 0.47)	0.73	0.95
Model 2	Referent	-0.30 (-1.39, 0.80)	-0.39 (-1.47, 0.69)	-0.34 (-1.44, 0.76)	0.90	0.94
**Flavanols**						
Model 1	Referent	0.03 (-0.74, 0.79)	0.26 (-0.53, 1.05)	0.41 (-0.41, 1.23)	0.72	0.95
Model 2	Referent	-0.62 (-1.70, 0.46)	-0.37 (-1.46, 0.71)	-0.13 (-1.25, 0.98)	0.68	0.94
**Flavanones**						
Model 1	Referent	0.28 (-0.46, 1.02)	0.11 (-0.64, 0.87)	0.03 (-0.74, 0.80)	0.88	0.95
Model 2	Referent	0.56 (-0.48, 1.61)	0.88 (-0.16, 1.93)	0.76 (-0.28, 1.81)	0.36	0.94
**Flavones**						
Model 1	Referent	0.43 (-0.31, 1.17)	0.32 (-0.43, 1.08)	0.22 (-0.55, 0.99)	0.71	0.95
Model 2	Referent	0.53 (-0.52, 1.59)	0.63 (-0.45, 1.70)	0.35 (-0.72, 1.42)	0.68	0.94
**Flavonols**						
Model 1	Referent	-0.64 (-1.40, 0.12)	-0.37 (-1.15, 0.42)	-0.47 (-1.29, 0.35)	0.42	0.95
Model 2	Referent	-1.07 (-2.14, 0.01)	-0.72 (-1.82, 0.37)	-0.81 (-1.92, 0.30)	0.26	0.94
**Isoflavonoids**						
Model 1	Referent	-0.73 (-1.47, 0.01)	-0.65 (-1.41, 0.10)	0.28 (-0.51, 1.06)	0.02	0.22
Model 2	Referent	-1.45 (-2.52, -0.37)	-1.05 (-2.12, 0.01)	-0.21 (-1.30, 0.88)	0.02	0.15
**Hydroxybenzoic acids**						
Model 1	Referent	-0.22 (-0.99, 0.56)	-0.19 (-0.99, 0.61)	-0.17 (-1.00, 0.67)	0.95	0.95
Model 2	Referent	-1.56 (-2.67, -0.45)	-1.58 (-2.70, -0.47)	-1.19 (-2.34, -0.05)	0.02	0.15
**Hydroxycinnamic acids**						
Model 1	Referent	-0.99 (-1.75, -0.24)	-0.97 (-1.76, -0.17)	-0.83 (-1.66, 0.00)	0.04	0.31
Model 2	Referent	-0.89 (-1.99, 0.22)	-0.68 (-1.80, 0.44)	-0.52 (-1.67, 0.62)	0.46	0.94

Model 1. Unadjusted for any covariate (N = 1,363)
Model 2. Adjusted for age, education, employment, physical activity, smoking status, and alcohol intake (N = 1,044); reduced sample size due to missing covariate data
[a] Adjusted P for overall difference using the Simes method

Table S25. Mean differences and 95% confidence intervals for the prospective association between energy-unadjusted intake of polyphenol subclasses and DASS depression subscale scores

Polyphenol subclass	Quartile 1	Quartile 2	Quartile 3	Quartile 4	P for overall difference	Q value [a]
**Anthocyanins**						
Median (IQR) (mg/day)	5.05 (3.21, 6.84)	12.21 (9.80, 14.39)	21.60 (17.33, 25.47)	45.84 (36.73, 63.16)		
Model 1	Referent	-0.57 (-1.31, 0.18)	-0.30 (-1.06, 0.47)	-0.84 (-1.62, -0.05)	0.17	0.29
Model 2	Referent	-0.55 (-1.61, 0.51)	-0.75 (-1.82, 0.32)	-1.03 (-2.12, 0.05)	0.30	0.47
**Flavanols**						
Median (IQR) (mg/day)	80.25 (59.13, 101.00)	160.56 (140.59, 180.75)	264.19 (233.18, 302.78)	489.21 (408.93, 630.07)		
Model 1	Referent	-0.75 (-1.49, -0.01)	-0.18 (-0.95, 0.60)	-0.21 (-1.02, 0.59)	0.21	0.32
Model 2	Referent	-1.75 (-2.78, -0.71)	-0.72 (-1.78, 0.34)	-1.04 (-2.13, 0.05)	0.01	0.07
**Flavanones**						
Median (IQR) (mg/day)	4.19 (2.14, 6.98)	12.99 (9.79, 17.57)	26.67 (20.56, 34.22)	57.92 (43.84, 76.96)		
Model 1	Referent	0.43 (-0.31, 1.18)	0.06 (-0.70, 0.82)	0.04 (-0.74, 0.82)	0.64	0.68
Model 2	Referent	0.48 (-0.57, 1.53)	0.86 (-0.20, 1.92)	0.44 (-0.64, 1.52)	0.47	0.55
**Flavones**						
Median (IQR) (mg/day)	2.71 (2.01, 3.37)	5.56 (4.80, 6.30)	9.28 (8.08, 11.20)	21.85 (16.54, 31.78)		
Model 1	Referent	-0.53 (-1.27, 0.21)	-0.08 (-0.84, 0.67)	-0.79 (-1.57, 0.00)	0.13	0.27
Model 2	Referent	-0.63 (-1.67, 0.42)	-0.11 (-1.17, 0.95)	-0.61 (-1.69, 0.47)	0.51	0.55
**Flavonols**						
Median (IQR) (mg/day)	14.59 (11.57, 17.12)	25.08 (21.28, 31.56)	40.86 (32.97, 47.84)	75.06 (59.66, 94.68)		
Model 1	Referent	-1.20 (-1.94, -0.47)	-0.70 (-1.47, 0.08)	-1.06 (-1.87, -0.25)	0.009	0.03
Model 2	Referent	-2.02 (-3.07, -0.98)	-1.30 (-2.36, -0.23)	-1.88 (-2.97, -0.78)	< 0.001	0.009
**Isoflavonoids**						
Median (IQR) (mg/day)	0.02 (0.01, 0.03)	0.11 (0.06, 0.26)	2.41 (1.67, 3.87)	11.44 (7.02, 19.92)		
Model 1	Referent	-0.05 (-0.80, 0.69)	-0.45 (-1.21, 0.31)	0.88 (0.10, 1.67)	0.004	0.03
Model 2	Referent	-0.52 (-1.58, 0.54)	-0.76 (-1.83, 0.30)	0.39 (-0.71, 1.49)	0.11	0.31
**Hydroxybenzoic acids**						
Median (IQR) (mg/day)	6.44 (4.39, 8.43)	15.39 (11.86, 18.77)	31.19 (25.88, 38.07)	80.48 (54.15, 102.47)		
Model 1	Referent	0.17 (-0.59, 0.94)	-0.18 (-0.97, 0.61)	-0.33 (-1.16, 0.50)	0.63	0.68
Model 2	Referent	-1.03 (-2.11, 0.05)	-1.70 (-2.81, -0.59)	-1.37 (-2.51, -0.23)	0.02	0.08

Polyphenol subclass	Quartile 1	Quartile 2	Quartile 3	Quartile 4	P for overall difference	Q value [a]
**Hydroxycinnamic acids**						
Median (IQR) (mg/day)	55.68 (39.85, 71.58)	144.76 (98.66, 285.66)	462.65 (281.11, 566.90)	1229.10 (832.36, 1315.38)		
Model 1	Referent	-1.01 (-1.76, -0.26)	-0.90 (-1.69, -0.10)	-0.86 (-1.69, -0.03)	0.04	0.10
Model 2	Referent	-0.72 (-1.80, 0.36)	-0.23 (-1.34, 0.88)	-0.26 (-1.36, 0.85)	0.61	0.61

IQR, interquartile range

Model 1. Unadjusted for any covariate (N = 1,484)

Model 2. Adjusted for age, sex, education, employment, physical activity, smoking status, and alcohol intake (N = 1,137); reduced sample size due to missing covariate data

[a] Adjusted P for overall difference using the Simes method

Association between polyphenol subclasses and depressive symptoms: Exploratory analyses

Table S26. Odds ratios (OR) and 95% confidence intervals (CI) for the prospective association between energy-adjusted intake of polyphenol subclasses and depression incidence

Polyphenol subclass	Quartile 1	Quartile 2	Quartile 3	Quartile 4	P for overall difference	Q value [a]
**Anthocyanins**						
Model 1 [OR (95% CI)]	Referent	0.77 (0.54, 1.11)	0.70 (0.48, 1.02)	0.67 (0.46, 0.98)	0.17	0.75
Model 2 [OR (95% CI)]	Referent	0.79 (0.47, 1.35)	0.62 (0.36, 1.06)	0.75 (0.44, 1.27)	0.37	0.79
**Flavanols**						
Model 1 [OR (95% CI)]	Referent	0.83 (0.57, 1.20)	1.03 (0.71, 1.50)	0.94 (0.64, 1.39)	0.64	0.82
Model 2 [OR (95% CI)]	Referent	0.64 (0.38, 1.10)	0.88 (0.52, 1.47)	0.81 (0.47, 1.37)	0.44	0.79
**Flavanones**						
Model 1 [OR (95% CI)]	Referent	1.22 (0.85, 1.74)	0.99 (0.68, 1.44)	1.02 (0.70, 1.49)	0.63	0.82
Model 2 [OR (95% CI)]	Referent	1.41 (0.85, 2.36)	1.18 (0.70, 1.99)	1.28 (0.76, 2.15)	0.60	0.79
**Flavones**						
Model 1 [OR (95% CI)]	Referent	1.13 (0.79, 1.62)	0.84 (0.58, 1.21)	0.91 (0.63, 1.32)	0.39	0.82
Model 2 [OR (95% CI)]	Referent	0.94 (0.56, 1.56)	0.77 (0.46, 1.31)	0.76 (0.45, 1.30)	0.67	0.79
**Flavonols**						
Model 1 [OR (95% CI)]	Referent	0.68 (0.47, 0.99)	0.83 (0.57, 1.21)	0.72 (0.49, 1.06)	0.18	0.75
Model 2 [OR (95% CI)]	Referent	0.45 (0.26, 0.77)	0.73 (0.43, 1.22)	0.51 (0.30, 0.88)	0.02	0.19
**Isoflavonoids**						
Model 1 [OR (95% CI)]	Referent	0.65 (0.45, 0.93)	0.72 (0.50, 1.04)	1.09 (0.76, 1.57)	0.01	0.17
Model 2 [OR (95% CI)]	Referent	0.48 (0.28, 0.81)	0.66 (0.39, 1.09)	0.91 (0.55, 1.51)	0.03	0.19
**Hydroxybenzoic acids**						
Model 1 [OR (95% CI)]	Referent	0.83 (0.57, 1.21)	1.02 (0.70, 1.48)	0.87 (0.59, 1.28)	0.64	0.82
Model 2 [OR (95% CI)]	Referent	0.49 (0.28, 0.86)	0.62 (0.36, 1.07)	0.57 (0.33, 0.99)	0.08	0.35

Polyphenol subclass	Quartile 1	Quartile 2	Quartile 3	Quartile 4	P for overall difference	Q value [a]
**Hydroxycinnamic acids**						
Model 1 [OR (95% CI)]	Referent	-0.40 (-0.77, -0.03)	-0.20 (-0.58, 0.18)	-0.18 (-0.56, 0.21)	0.21	0.75
Model 2 [OR (95% CI)]	Referent	-0.35 (-0.90, 0.20)	-0.07 (-0.61, 0.48)	0.07 (-0.47, 0.60)	0.44	0.79

Model 1. Unadjusted for any covariate (N = 1,484)

Model 2. Adjusted for age, education, employment, physical activity, smoking status, and alcohol intake (N = 1,137); reduced sample size due to missing covariate data

[a] Adjusted P for overall difference using the Simes method

Table S27. Mean differences and 95% confidence intervals for the prospective association between energy-adjusted intake of polyphenol subclasses and total DASS score

Polyphenol subclass	Quartile 1	Quartile 2	Quartile 3	Quartile 4	P for overall difference	Q value [a]
**Anthocyanins**						
Model 1	Referent	-0.66 (-2.47, 1.15)	-0.37 (-2.21, 1.47)	-0.10 (-2.00, 1.79)	0.89	0.94
Model 2	Referent	-0.67 (-3.28, 1.95)	-0.79 (-3.39, 1.81)	-1.17 (-3.81, 1.47)	0.86	0.97
**Flavanols**						
Model 1	Referent	0.74 (-1.07, 2.54)	0.64 (-1.23, 2.51)	1.68 (-0.28, 3.64)	0.40	0.94
Model 2	Referent	-0.83 (-3.41, 1.76)	-0.89 (-3.48, 1.71)	-0.28 (-2.97, 2.42)	0.88	0.97
**Flavanones**						
Model 1	Referent	0.40 (-1.35, 2.15)	-0.02 (-1.83, 1.78)	-0.15 (-1.98, 1.69)	0.94	0.94
Model 2	Referent	1.06 (-1.45, 3.57)	0.81 (-1.73, 3.35)	1.28 (-1.26, 3.82)	0.77	0.97
**Flavones**						
Model 1	Referent	1.15 (-0.60, 2.90)	0.76 (-1.03, 2.55)	0.11 (-1.72, 1.95)	0.54	0.94
Model 2	Referent	0.52 (-2.01, 3.05)	0.65 (-1.93, 3.22)	-0.07 (-2.67, 2.52)	0.93	0.97
**Flavonols**						
Model 1	Referent	-1.79 (-3.57, 0.00)	-0.35 (-2.22, 1.52)	-0.51 (-2.46, 1.44)	0.21	0.70
Model 2	Referent	-3.83 (-6.39, -1.27)	-1.56 (-4.20, 1.08)	-2.42 (-5.09, 0.26)	0.03	0.27
**Isoflavonoids**						
Model 1	Referent	-1.22 (-2.96, 0.51)	-1.10 (-2.89, 0.70)	0.97 (-0.90, 2.84)	0.05	0.26
Model 2	Referent	-3.03 (-5.58, -0.48)	-2.11 (-4.66, 0.44)	-0.04 (-2.66, 2.57)	0.04	0.27
**Hydroxybenzoic acids**						
Model 1	Referent	-0.05 (-1.88, 1.77)	0.48 (-1.41, 2.37)	0.88 (-1.11, 2.87)	0.77	0.94
Model 2	Referent	-1.75 (-4.41, 0.91)	-3.10 (-5.78, -0.42)	-1.34 (-4.09, 1.40)	0.15	0.69
**Hydroxycinnamic acids**						
Model 1	Referent	-1.91 (-3.70, -0.11)	-1.35 (-3.24, 0.55)	0.30 (-1.68, 2.28)	0.04	0.26
Model 2	Referent	-1.15 (-3.81, 1.51)	-0.86 (-3.57, 1.85)	0.98 (-1.76, 3.71)	0.34	0.97

Model 1. Unadjusted for any covariate (N = 1,483)

Model 2. Adjusted for age, education, employment, physical activity, smoking status, and alcohol intake (N = 1,135); reduced sample size due to missing covariate data

[a] Adjusted P for overall difference using the Simes method

Table S28. Mean differences and 95% confidence intervals for the prospective association between energy-adjusted intake of polyphenol subclasses and DASS depression subscale scores after excluding polyphenols from alcohol

**Polyphenol subclass**	**Quartile 1**	**Quartile 2**	**Quartile 3**	**Quartile 4**	**P for overall difference**	**Q value [a]**
**Anthocyanins**						
Energy-adjusted (mg/day) [b] Median (IQR)	-13.94 (-16.67, -11.94)	-7.70 (-9.05, -6.35)	-1.47 (-3.03, 0.58)	15.71 (8.52, 28.20)		
Energy-unadjusted (mg/day) [c] Median (IQR)	5.26 (2.92, 7.80)	8.59 (5.73, 12.30)	15.47 (11.20, 20.07)	35.10 (26.08, 48.63)		
Model 1	Referent	-0.46 (-1.22, 0.29)	-0.33 (-1.10, 0.44)	-0.89 (-1.67, -0.11)	0.15	0.54
Model 2	Referent	-0.19 (-1.27, 0.89)	-0.12 (-1.22, 0.98)	-0.46 (-1.55, 0.64)	0.86	0.93
**Flavanols**						
Energy-adjusted (mg/day) [b] Median (IQR)	-166.55 (-197.53, -150.12)	-97.84 (-115.40, -77.26)	-5.66 (-32.86, 24.36)	213.00 (131.49, 340.45)		
Energy-unadjusted (mg/day) [c] Median (IQR)	77.08 (55.62, 108.68)	146.57 (115.91, 176.87)	244.61 (208.18, 286.42)	471.01 (384.86, 609.79)		
Model 1	Referent	-0.17 (-0.93, 0.59)	0.16 (-0.61, 0.94)	0.08 (-0.73, 0.89)	0.84	0.95
Model 2	Referent	-0.43 (-1.51, 0.64)	-0.03 (-1.10, 1.04)	-0.35 (-1.46, 0.76)	0.80	0.93
**Flavanones**						
Energy-adjusted (mg/day) [b] Median (IQR)	-21.16 (-26.21, -17.37)	-11.84 (-14.56, -9.90)	-1.15 (-4.42, 3.33)	25.07 (15.32, 43.72)		
Energy-unadjusted (mg/day) [c] Median (IQR)	5.53 (2.30, 10.04)	10.66 (6.41, 16.53)	24.04 (16.89, 32.73)	55.95 (40.88, 75.56)		
Model 1	Referent	0.10 (-0.64, 0.84)	0.02 (-0.74, 0.77)	-0.17 (-0.93, 0.60)	0.92	0.95
Model 2	Referent	0.11 (-0.94, 1.17)	0.61 (-0.43, 1.65)	0.40 (-0.64, 1.45)	0.65	0.93

Polyphenol subclass	Quartile 1	Quartile 2	Quartile 3	Quartile 4	P for overall difference	Q value [a]
**Flavones**						
Energy-adjusted (mg/day) [b] Median (IQR)	-7.80 (-10.30, -6.33)	-3.94 (-4.71, -3.23)	-0.60 (-1.63, 0.65)	9.65 (5.92, 17.28)		
Energy-unadjusted (mg/day) [c] Median (IQR)	4.25 (2.71, 6.83)	4.85 (3.24, 6.62)	7.61 (5.48, 10.11)	20.82 (14.94, 31.35)		
Model 1	Referent	0.27 (-0.46, 1.01)	0.07 (-0.68, 0.82)	-0.06 (-0.83, 0.70)	0.83	0.95
Model 2	Referent	0.23 (-0.82, 1.28)	0.43 (-0.64, 1.49)	-0.04 (-1.11, 1.04)	0.81	0.93
**Flavonols**						
Energy-adjusted (mg/day) [b] Median (IQR)	-23.13 (-27.78, -19.75)	-13.43 (-15.91, -11.14)	-1.68 (-5.32, 3.13)	29.64 (17.17, 50.35)		
Energy-unadjusted (mg/day) [c] Median (IQR)	14.43 (11.03, 19.06)	23.96 (19.13, 30.64)	37.48 (29.82, 46.32)	71.64 (56.18, 91.91)		
Model 1	Referent	-0.91 (-1.66, -0.16)	-0.56 (-1.34, 0.22)	-0.86 (-1.67, -0.05)	0.08	0.38
Model 2	Referent	-1.26 (-2.33, -0.20)	-0.69 (-1.78, 0.40)	-1.36 (-2.47, -0.25)	0.05	0.36
**Isoflavonoids**						
Energy-adjusted (mg/day) [b] Median (IQR)	-6.40 (-6.94, -4.14)	-5.77 (-6.17, -3.87)	-2.64 (-3.83, -1.58)	5.80 (1.67, 14.18)		
Energy-unadjusted (mg/day) [c] Median (IQR)	0.03 (0.01, 0.05)	0.08 (0.04, 0.34)	2.40 (1.66, 3.87)	11.44 (7.01, 19.89)		
Model 1	Referent	-0.67 (-1.40, 0.05)	-0.65 (-1.40, 0.10)	0.39 (-0.39, 1.17)	0.010	0.13
Model 2	Referent	-1.46 (-2.51, -0.41)	-1.17 (-2.22, -0.12)	-0.23 (-1.31, 0.84)	0.01	0.18
**Hydroxybenzoic acids**						
Energy-adjusted (mg/day) [b] Median (IQR)	-17.32 (-19.25, -13.92)	-11.45 (-14.06, -8.78)	-2.97 (-6.02, 0.16)	19.90 (10.28, 38.51)		
Energy-unadjusted (mg/day) [c] Median (IQR)	5.29 (3.49, 7.26)	12.39 (8.79, 15.97)	27.49 (21.66, 34.65)	73.62 (48.31, 96.80)		
Model 1	Referent	-0.09 (-0.84, 0.65)	-0.27 (-1.05, 0.52)	-0.54 (-1.36, 0.27)	0.58	0.82
Model 2	Referent	-0.41 (-1.48, 0.65)	-1.07 (-2.15, 0.01)	-1.11 (-2.21, -0.01)	0.14	0.67

Polyphenol subclass	Quartile 1	Quartile 2	Quartile 3	Quartile 4	P for overall difference	Q value [a]
**Hydroxycinnamic acids**						
Energy-adjusted (mg/day) [b] Median (IQR)	-249.07 (-282.10, -196.28)	-152.32 (-181.81, -128.48)	-31.06 (-66.53, 21.80)	311.07 (204.47, 496.31)		
Energy-unadjusted (mg/day) [c] Median (IQR)	55.43 (38.95, 73.13)	144.98 (97.36, 281.70)	467.12 (278.11, 571.86)	1205.18 (820.36, 1295.41)		
Model 1	Referent	-0.91 (-1.67, -0.16)	-0.99 (-1.78, -0.20)	-0.61 (-1.43, 0.22)	0.05	0.37
Model 2	Referent	-0.75 (-1.85, 0.34)	-0.66 (-1.78, 0.45)	-0.20 (-1.33, 0.93)	0.46	0.93

IQR, interquartile range

Model 1. Unadjusted for any covariate (N = 1,484)

Model 2. Adjusted for age, education, employment, physical activity, smoking status, and alcohol intake (N = 1,137); reduced sample size due to missing covariate data

[a] Adjusted P for overall difference using the Simes method

[b] Energy-adjusted values derived using Willett's residual method

[c] Energy-unadjusted values reflect intakes within energy-adjusted polyphenol intake quartiles

Association between polyphenol subclasses and depressive symptoms: Subgroup analyses

Table S29. Mean differences and 95% confidence intervals for the prospective association between energy-adjusted intake of polyphenol subclasses and DASS depression subscale scores by smoking status at baseline

Polyphenol subclass	Smoking status	Quartile 1	Quartile 2	Quartile 3	Quartile 4	P for overall difference	Q value [a]
**Anthocyanins**	Non-smoker	Referent	0.40 (-0.73, 1.53)	-0.01 (-1.14, 1.12)	-0.64 (-1.81, 0.52)	0.32	0.75
	Current smoker	Referent	-1.20 (-4.19, 1.80)	-0.89 (-4.07, 2.30)	1.63 (-1.74, 5.00)	0.31	0.63
**Flavanols**	Non-smoker	Referent	-0.21 (-1.34, 0.92)	0.23 (-0.91, 1.37)	-0.25 (-1.44, 0.95)	0.80	0.84
	Current smoker	Referent	-1.61 (-4.63, 1.41)	0.13 (-3.08, 3.34)	3.83 (0.62, 7.03)	0.02	0.17
**Flavanones**	Non-smoker	Referent	0.47 (-0.59, 1.53)	0.53 (-0.57, 1.64)	0.12 (-0.99, 1.22)	0.72	0.84
	Current smoker	Referent	3.61 (0.58, 6.65)	3.89 (0.81, 6.98)	1.68 (-1.39, 4.74)	0.05	0.18
**Flavones**	Non-smoker	Referent	0.68 (-0.41, 1.78)	0.39 (-0.73, 1.50)	-0.04 (-1.16, 1.09)	0.51	0.84
	Current smoker	Referent	-0.87 (-3.68, 1.94)	2.11 (-0.88, 5.11)	1.05 (-2.34, 4.44)	0.20	0.46
**Flavonols**	Non-smoker	Referent	-0.71 (-1.82, 0.40)	-0.29 (-1.44, 0.86)	-1.12 (-2.30, 0.06)	0.23	0.72
	Current smoker	Referent	0.32 (-2.66, 3.29)	0.74 (-2.26, 3.74)	4.19 (1.04, 7.34)	0.04	0.17
**Isoflavonoids**	Non-smoker	Referent	-0.84 (-1.87, 0.20)	-0.63 (-1.70, 0.45)	0.52 (-0.61, 1.66)	0.05	0.71
	Current smoker	Referent	0.32 (-2.89, 3.53)	-0.58 (-3.76, 2.60)	-0.44 (-3.81, 2.92)	0.94	0.99
**Hydroxybenzoic acids**	Non-smoker	Referent	-0.85 (-1.96, 0.26)	-0.76 (-1.93, 0.41)	-0.93 (-2.13, 0.26)	0.38	0.77
	Current smoker	Referent	-1.06 (-4.51, 2.38)	-0.06 (-3.41, 3.29)	1.38 (-1.98, 4.74)	0.56	0.97
**Hydroxycinnamic acids**	Non-smoker	Referent	-0.88 (-1.97, 0.22)	-1.12 (-2.28, 0.03)	-0.76 (-1.95, 0.43)	0.26	0.72
	Current smoker	Referent	-0.91 (-4.34, 2.52)	-1.14 (-4.68, 2.40)	-0.05 (-3.55, 3.46)	0.84	0.99

Adjusted for age, education, employment, physical activity, and alcohol intake
Non-smoker (N = 737)
Current smoker (N = 110)
[a] Adjusted P for overall difference using the Simes method

Table S30. Mean differences and 95% confidence intervals for the prospective association between energy-adjusted intake of polyphenol subclasses and DASS depression subscale scores by physical activity status at baseline

Polyphenol subclass	Physical activity guidelines	Quartile 1	Quartile 2	Quartile 3	Quartile 4	P for overall difference	Q value [a]
**Anthocyanins**	Not met	Referent	-0.04 (-2.45, 2.38)	-1.68 (-4.08, 0.71)	-1.17 (-3.66, 1.31)	0.43	0.65
	Met	Referent	0.33 (-1.05, 1.71)	0.15 (-1.22, 1.52)	0.11 (-1.31, 1.54)	0.97	0.97
**Flavanols**	Not met	Referent	-1.92 (-4.27, 0.44)	-2.67 (-5.03, -0.31)	-0.97 (-3.48, 1.54)	0.13	0.65
	Met	Referent	0.85 (-0.52, 2.21)	1.16 (-0.22, 2.54)	0.80 (-0.65, 2.25)	0.42	0.65
**Flavanones**	Not met	Referent	0.19 (-2.20, 2.58)	-1.15 (-3.55, 1.26)	-0.40 (-2.80, 2.00)	0.69	0.72
	Met	Referent	0.73 (-0.57, 2.03)	1.23 (-0.10, 2.56)	0.72 (-0.63, 2.06)	0.34	0.65
**Flavones**	Not met	Referent	-0.53 (-2.99, 1.92)	-1.41 (-3.96, 1.14)	-0.97 (-3.38, 1.45)	0.72	0.72
	Met	Referent	1.48 (0.17, 2.79)	1.80 (0.48, 3.12)	0.99 (-0.39, 2.38)	0.05	0.64
**Flavonols**	Not met	Referent	-2.52 (-4.86, -0.19)	-0.93 (-3.30, 1.44)	-1.23 (-3.73, 1.26)	0.20	0.65
	Met	Referent	-0.10 (-1.43, 1.24)	-0.13 (-1.53, 1.27)	-0.45 (-1.87, 0.97)	0.93	0.97
**Isoflavonoids**	Not met	Referent	-1.45 (-3.66, 0.77)	-1.89 (-4.29, 0.51)	-0.48 (-2.90, 1.95)	0.36	0.65
	Met	Referent	-1.23 (-2.57, 0.12)	-0.90 (-2.22, 0.41)	-0.09 (-1.50, 1.31)	0.19	0.64
**Hydroxybenzoic acids**	Not met	Referent	-2.46 (-5.06, 0.13)	-1.77 (-4.22, 0.68)	-2.07 (-4.53, 0.39)	0.24	0.65
	Met	Referent	-1.08 (-2.45, 0.29)	-1.36 (-2.75, 0.04)	-0.97 (-2.42, 0.48)	0.27	0.64
**Hydroxycinnamic acids**	Not met	Referent	-1.04 (-3.41, 1.32)	-2.23 (-4.80, 0.34)	-0.27 (-2.82, 2.28)	0.30	0.65
	Met	Referent	-0.77 (-2.14, 0.60)	-0.80 (-2.23, 0.64)	-0.59 (-2.05, 0.87)	0.68	0.89

Adjusted for age, education, employment, smoking status, and alcohol intake
Not met (N = 223)
Met (N = 600)
[a] Adjusted P for overall difference using the Simes method

Table S31. Mean differences and 95% confidence intervals for the prospective association between energy-adjusted intake of polyphenol subclasses and DASS depression subscale scores by alcohol consumption at baseline

Polyphenol subclass	Alcohol consumption	Quartile 1	Quartile 2	Quartile 3	Quartile 4	P for overall difference	Q value [a]
**Anthocyanins**	Nil to low	Referent	0.00 (-1.54, 1.54)	-0.74 (-2.27, 0.79)	-1.29 (-2.87, 0.29)	0.29	0.63
	Moderate to high	Referent	-0.19 (-2.34, 1.96)	1.28 (-0.83, 3.39)	2.21 (0.04, 4.37)	0.10	0.35
**Flavanols**	Nil to low	Referent	-0.93 (-2.47, 0.60)	-1.03 (-2.55, 0.49)	-1.29 (-2.90, 0.32)	0.43	0.67
	Moderate to high	Referent	0.14 (-2.02, 2.30)	1.91 (-0.30, 4.11)	2.14 (-0.07, 4.34)	0.13	0.35
**Flavanones**	Nil to low	Referent	0.27 (-1.20, 1.75)	0.12 (-1.40, 1.64)	0.56 (-0.96, 2.08)	0.89	0.94
	Moderate to high	Referent	1.45 (-0.57, 3.47)	1.89 (-0.16, 3.95)	0.13 (-1.90, 2.16)	0.18	0.35
**Flavones**	Nil to low	Referent	0.81 (-0.71, 2.32)	0.54 (-1.02, 2.10)	-0.37 (-1.96, 1.22)	0.39	0.67
	Moderate to high	Referent	-0.11 (-2.20, 1.98)	1.15 (-0.85, 3.16)	1.07 (-0.96, 3.10)	0.48	0.56
**Flavonols**	Nil to low	Referent	-1.27 (-2.75, 0.21)	-0.66 (-2.20, 0.89)	-1.80 (-3.36, -0.23)	0.11	0.53
	Moderate to high	Referent	-0.32 (-2.50, 1.86)	1.00 (-1.24, 3.25)	1.71 (-0.53, 3.96)	0.26	0.41
**Isoflavonoids**	Nil to low	Referent	-1.38 (-2.83, 0.07)	-1.05 (-2.49, 0.38)	-0.42 (-1.98, 1.13)	0.24	0.63
	Moderate to high	Referent	-1.22 (-3.45, 1.00)	-0.52 (-2.84, 1.81)	0.33 (-1.94, 2.61)	0.46	0.56
**Hydroxybenzoic acids**	Nil to low	Referent	-1.85 (-3.35, -0.34)	-1.33 (-2.86, 0.20)	-1.86 (-3.39, -0.32)	0.05	0.37
	Moderate to high	Referent	-0.97 (-3.39, 1.45)	-0.67 (-3.04, 1.71)	0.97 (-1.50, 3.44)	0.27	0.41
**Hydroxycinnamic acids**	Nil to low	Referent	-1.10 (-2.52, 0.33)	-1.05 (-2.61, 0.51)	0.16 (-1.43, 1.75)	0.20	0.63
	Moderate to high	Referent	0.83 (-1.79, 3.46)	0.59 (-1.79, 2.96)	0.33 (-2.08, 2.74)	0.93	0.93

Adjusted for age, education, employment, physical activity, and smoking status

Nil to low (N = 524)

Moderate to high (N = 242)

[a] Adjusted P for overall difference using the Simes method

Table S32. Mean differences and 95% confidence intervals for the prospective association between energy-adjusted intake of polyphenol subclasses and DASS depression subscale scores by sex

Polyphenol subclass	Alcohol consumption	Quartile 1	Quartile 2	Quartile 3	Quartile 4	P for overall difference	Q value [a]
**Anthocyanins**	Male	Referent	0.40 (-0.98, 1.78)	0.06 (-1.33, 1.46)	1.00 (-0.43, 2.43)	0.53	0.69
	Female	Referent	-1.74 (-3.44, -0.04)	-1.79 (-3.46, -0.12)	-2.36 (-4.05, -0.67)	0.05	0.37
**Flavanols**	Male	Referent	-0.30 (-1.72, 1.11)	0.82 (-0.59, 2.22)	0.63 (-0.91, 2.16)	0.42	0.69
	Female	Referent	-1.14 (-2.77, 0.48)	-1.44 (-3.08, 0.20)	-1.67 (-3.31, -0.02)	0.24	0.41
**Flavanones**	Male	Referent	0.14 (-1.28, 1.56)	0.48 (-0.95, 1.91)	-0.62 (-2.02, 0.77)	0.49	0.69
	Female	Referent	1.24 (-0.25, 2.73)	1.14 (-0.38, 2.66)	1.71 (0.15, 3.27)	0.17	0.41
**Flavones**	Male	Referent	-0.17 (-1.57, 1.23)	1.15 (-0.30, 2.61)	0.03 (-1.30, 1.35)	0.34	0.69
	Female	Referent	0.00 (-1.64, 1.63)	-0.40 (-2.04, 1.24)	-0.35 (-2.10, 1.41)	0.92	0.92
**Flavonols**	Male	Referent	-1.18 (-2.58, 0.22)	-1.11 (-2.58, 0.36)	-0.45 (-1.96, 1.07)	0.31	0.69
	Female	Referent	-1.88 (-3.46, -0.30)	-0.83 (-2.44, 0.77)	-2.15 (-3.76, -0.54)	0.02	0.33
**Isoflavonoids**	Male	Referent	-1.79 (-3.30, -0.28)	-1.23 (-2.81, 0.36)	-0.64 (-2.19, 0.92)	0.10	0.47
	Female	Referent	-0.87 (-2.38, 0.64)	-1.01 (-2.43, 0.40)	0.35 (-1.17, 1.86)	0.20	0.41
**Hydroxybenzoic acids**	Male	Referent	-1.69 (-3.15, -0.23)	-1.48 (-3.03, 0.07)	-0.61 (-2.31, 1.10)	0.10	0.47
	Female	Referent	-1.24 (-2.87, 0.39)	-1.46 (-3.02, 0.10)	-1.64 (-3.16, -0.13)	0.16	0.41
**Hydroxycinnamic acids**	Male	Referent	-1.17 (-2.77, 0.43)	-0.83 (-2.49, 0.83)	-0.99 (-2.69, 0.70)	0.54	0.69
	Female	Referent	-0.57 (-2.09, 0.95)	-0.36 (-1.89, 1.16)	0.37 (-1.14, 1.87)	0.64	0.74

Adjusted for age, education, employment, physical activity, smoking status, and alcohol intake
Male (N = 541)
Female (N = 596)
[a] Adjusted P for overall difference using the Simes method
